# Use of Hyperspectral Imagery to Assess Cryptic Color Matching in *Sargassum* Associated Crabs

**DOI:** 10.1371/journal.pone.0136260

**Published:** 2015-09-09

**Authors:** Brandon J. Russell, Heidi M. Dierssen

**Affiliations:** 1 Department of Marine Science, University of Connecticut, Groton, CT, 06340, United States of America; 2 Department of Geography, University of Connecticut, Storrs, CT, 06268, United States of America; Lund University, SWEDEN

## Abstract

Mats of the pelagic macroalgae *Sargassum* represent a complex environment for the study of marine camouflage at the air-sea interface. Endemic organisms have convergently evolved similar colors and patterns, but quantitative assessments of camouflage strategies are lacking. Here, spectral camouflage of two crab species (*Portunus sayi* and *Planes minutus*) was assessed using hyperspectral imagery (HSI). Crabs matched *Sargassum* reflectance across blue and green wavelengths (400–550 nm) and diverged at longer wavelengths. Maximum discrepancy was observed in the far-red (i.e., 675 nm) where Chlorophyll *a* absorption occurred in *Sargassum* and not the crabs. In a quantum catch color model, both crabs showed effective color matching against blue/green sensitive dichromat fish, but were still discernible to tetrachromat bird predators that have visual sensitivity to far red wavelengths. The two species showed opposing trends in background matching with relation to body size. Variation in model parameters revealed that discrimination of crab and background was impacted by distance from the predator, and the ratio of cone cell types for bird predators. This is one of the first studies to detail background color matching in this unique, challenging ecosystem at the air-sea interface.

## Introduction

The need to hide is a potent evolutionary driver [1]. Biological camouflage, the ability to avoid detection or recognition by an observer, has evolved in multiple phyla as an adaptation to visually-orienting predators and prey. The study of marine camouflage systems has relevance to ecology and animal behavior as well as human naval operations, and to target discrimination in the water column [2, 3]. A wide variety of strategies exist, and the classification of camouflage techniques is a complex field [4]. One of the central topics of camouflage is color—Does an animal match the color of its background? The concept of general background color matching has been integral to the study of camouflage throughout its history, and this strategy is common in the marine environment [1, 4–11].

Early camouflage studies were typically biased towards human vision and subjective color assessment [4]. The use of calibrated Red-Green-Blue (RGB) and other forms of photography has effectively been applied to study color patterns and background matching [12–18], though it does not provide fully spectrally resolved light data. Spectroradiometry with fiber optic probes has been widely used in terrestrial and marine camouflage research to provide full spectral reflectance from an organism and address color discrimination by observers [8, 11, 19–22]. For animals and backgrounds which have small, highly contrasting color elements, however, sampling of the entire pattern with a spectroradiometer probe is difficult and provides isolated data points that may not always be statistically representative of the entire organism without equipment modification and rigorous technique [21]. An additional sampling concern is contamination of the signal from specular reflectance. While this can be greatly minimized through proper alignment of subject, light, and probe [20], it remains a concern particularly on smooth, curved animal surfaces like those under investigation here.

Hyperspectral imagery (HSI) represents a combination of these traditional techniques and is an invaluable tool for the objective study of spectral camouflage [23–25]. A hyperspectral imager provides a near-synoptic image with full spectral or “hyperspectral” information for every pixel covering an organism and its background habitat. Specular reflection can be easily identified and removed from the dataset. Moreover, the large volume of gridded data allows for statistical processing of both the spectral and spatial components of animal and background patterns which are impossible or difficult to sample with other methods [26]. Recent developments in highly portable imagers allow the use of hyperspectral imagery in the field and onboard small vessels to investigate dynamic changes in animal coloration. Here, a portable imager was used to investigate camouflage in two species of crab endemic to floating mats of the brown macroalgae *Sargassum* (*S*. *natans* and *S*. *fluitans*).

*Sargassum* mats cover vast areas of the ocean surface in the subtropical North Atlantic [27–29]. These mats serve as important primary producers [30, 31] and habitat in the open ocean. Several hundred species are known to associate with or utilize the mats, including an endemic faunal community [27, 32–36]. A *Sargassum* mat is a complex optical and structural environment. Mats contain both pelagic species in varying stages of age and biofouling, as well as other biological and artificial debris. Organisms adapted to this habitat have evolved a high degree of crypsis. There is no hard cover in which to hide, and animals may be detected simultaneously from any angle. Color and patterning which mimic the algae are common among endemics, as is the use of shapes which resemble fronds, stipes, or gas vesicles [37–39]. Animals seeking refuge within *Sargassum* are under threat of detection from multiple classes of visual system with differing spectral sensitivities. Important predators in the mats include several species of seabird [40], pelagic fish [31, 32, 41], and *Sargassum* endemics including the frogfish *Histrio histrio* and swimming crab *Portunus sayi* [27, 31, 42, 43]. Research on camouflage and predator avoidance in this environment has focused on crustaceans, which are among the most abundant *Sargassum* animals by species, number, and biomass [27]. The present study builds from pioneering work conducted 70–100 years ago focusing on chromatophore response to background in *Sargassum* crabs and shrimp [5, 9, 37], but employs the use of hyperspectral imagery to quantify the ability of predators to distinguish between the crab and its background based on color. As outlined in [6] and [26], quantitative studies of camouflage must consider three main factors: 1) the reflectance properties of the target and background, 2) the ambient light field and optical environment, and 3) the visual system of the observer. While multiple studies have investigated background matching under varying light fields [6, 19, 21, 22] and predator visual systems [11, 21, 23], this is one of the first studies to use hyperspectral imagery to investigate all of these components of spectral matching and crypsis in terms of predation from both in-water and airborne predators.

## Methods

This study focused on camouflage of two different species of crab collected from the Sargasso Sea. The methods for capturing organisms, measuring the hyperspectral reflectance properties of crabs and background *Sargassum*, quantifying the ambient light field and optical environment, and for assessing background color matching from the perspective of different predators are discussed below.

### 2.1 Collection of Crabs and *Sargassum*

Organisms and spectra were collected aboard the sailing vessel *Sea Dragon* on two cruises (26–28 May 2013 and 2–5 June 2013) south of Bermuda in the vicinity of 31° 12.29'N, 64° 40.41'W. Collection took place under the auspices and protocols of the Bermuda Natural History Museum as part of a larger research effort. No specific permits were required for collection of *Sargassum* or associated invertebrates, and no protected or endangered species were collected. Samples of *Sargassum sp*. were collected using dip nets with hole size < 3mm [27, 33, 39]. *Sargassum* collections were hand-sorted in buckets of seawater on deck shortly after sampling. Crabs were separated into individual containers with water and placed in shade. Samples of the *Sargassum* immediately surrounding the crabs when found were also taken (approximately 300mL) in an attempt to identify the microhabitat in which the individual was located just prior to collection, i.e. the specific background it would have naturally been viewed against. When this was not possible due to displacement of an individual within the net, a random sample from within that collection was taken. Crabs and macroalgae were quickly imaged (typically < 30 minutes) to prevent possible effects from thermal or other stressors. Animals were returned to the water with live algae samples after imaging.

### 2.2 Hyperspectral Imaging and Analysis

Spectral information was collected using a tripod-mounted 710 Hyperspectral Imager (Surface Optics Corporation). This instrument collects an image (520 x 696 pixels) with spectral information at 128 bands with 5 nm spacing from 380 nm to1040 nm, known as a “data cube.” Study organisms were gently restrained and imaged under daylight illumination on a diffusive, matte dark grey background (average *R* = 0.12) at approximately normal incidence. Every image included a Spectralon (LabSphere) standard for calculation of reflectance *R*(λ), the ratio of incident photons scattered backwards off the target at each wavelength [44]. The calculated reflectance is independent of the light field and considered to be an inherent optical property of the target. For this study, the target was assumed to be Lambertian, reflecting light equally at all angles. While the surfaces of crabs and algae are most likely non-Lambertian, our assumption is reasonable when applied to the element of reflectance which includes color information (as opposed to glare or specular reflection off the carapace surface) [20] and was sampled by our approach.

ENVI (Exelis VIS) image analysis software was used to process data cubes. All spectra were interpolated from instrument and standard calibrations to 1 nm resolution. The image was converted to reflectance by normalizing to an average radiance obtained from non-saturated pixels centered on the Spectralon standard (generally >500 pixels). For each individual crab, Regions of Interest (ROIs) were hand selected to include the carapace surface and avoid visibly glare-contaminated pixels. Saturated pixels were identified and masked. Algae pixels were extracted from the imagery using a Normalized Difference Vegetation Index (NDVI), which contrasts reflectance in near infrared wavelengths (700 nm) to red wavelengths (670 nm) and has been previously used to identify *Sargassum* [44]. The NDVI identifies algal pixels containing a “red edge,” the sharp rise in reflectance in far red wavelengths common to plant material. Pixels with calcareous epibionts are not identified with an NDVI and were selected manually. For the statistical analyses, a random subset of *Sargassum* pixels equal to the number of available crab carapace pixels (~500–1500 pixels) was selected as representative of the background for that individual. Mean reflectance curves for each individual crab and corresponding algal background were then generated.

### 2.3 Discrimination by Predators

Color vision in vertebrates is the result of input from multiple classes of photoreceptive cone cell viewing the same visual field, each of which is sensitive to a different range of wavelengths [45]. The difference in signal from the receptor classes is then compared via an opponency mechanism to produce the sensation of color. Systems with two, three, and four classes are classified as di, tri, and tetrachromats, respectively [6, 45, 46]. To assess whether two colored targets might be distinguishable/discriminable to a given observer under given light conditions, the widely used Vorobyev and Osorio model [46] was applied. This model was used to investigate color matching in the view of two generalized predator types: fish and birds. Dichromat piscine (mahi mahi—*Coryphaena hippurus*) and tetrachromat avian (wedge-tailed shearwater—*Puffinus pacificus)* models were selected. *C*. *hippurus*, a blue/green sensitive pelagic fish [47, 48], is a frequent *Sargassum* visitor shown to consume *P*. *sayi* through gut content analysis [43] and is one of the only known predators of *Sargassum* crabs for which spectral sensitivity data is available. Multiple taxa of sea birds forage for fish and crustaceans in *Sargassum* lines [40]. *P*. *pacificus* was selected for visual modeling as it is perhaps the most comprehensively studied for spectral sensitivity in marine birds [49, 50], and it is broadly sensitive to wavelengths from the ultraviolet to far red.

To estimate the color contrast of crabs and algae to the predator visual system, the proportion of incident photons captured by each class or type of photoreceptor (quantum catch) was first calculated for each crab and algae spectrum according to the general form:

$$\begin{array}{l} q_{i}=k_{i}\overset{\lambda max}{\underset{\lambda min}{\int }}I\left(\lambda \right)T\left(\lambda \right)R\left(\lambda \right)S_{i}\left(\lambda \right)d\lambda \\ k_{i}=1÷\overset{\lambda max}{\underset{\lambda min}{\int }}I\left(\lambda \right)T\left(\lambda \right)R^{b}\left(\lambda \right)S_{i}\left(\lambda \right)d\lambda \\ Q_{i}=\text{ln}\;q_{i} \end{array}$$ (1)

where *λ*_*min*_ and *λ*_*max*_ are the limits of the spectral region considered, *i* is the photoreceptor type (with higher numbers indicating longer *λ* sensitivity), *q*_*i*_ is the receptor quantum catch for a given photoreceptor type, *k*_*i*_ is the von Kries transformation for color constancy, *T*(λ) is light transmission between target and observer, *R*(λ) is the reflectance of a target, *R* ^*b*^(λ) is the mean reflectance of the visual field, *S*_*i*_(λ) is the spectral sensitivity of the receptor, and finally *Q*_*i*_ is the coded, logarithmic quantum catch for that photoreceptor [11, 19, 23 46]. The lower bound, *λ*_*min*_, was set at 400 nm due to the signal-to-noise limitation of the imager, while *λ*_*max*_, 700 nm, represents the upper boundary of light sensitivity in the bird model. Photopigment absorption spectra *S*(λ) for each predator were taken from published sources. Fish sensitivity spectra [47] were digitized and a transmission curve (T50 = 436nm [48]) was applied to produce final sensitivity spectra *S*_*i*_. Bird spectral sensitivities [50, 51] were digitized and directly utilized in the model.

In this chromatic discrimination model, contrast between crab and algae quantum catch signals for each photoreceptor type are determined via a neural opponency mechanism [22, 23, 46]. It is assumed that detection is limited by physiological noise in the receptor channels themselves. Similarity between two target colors was calculated as chromatic contrast (ΔS).

For the fish predator:

$$\Delta S^{2}\;=\;{\left(\Delta Q_{1}-\Delta Q_{2}\right)}^{2}/\left(e_{1}^{2}+e_{2}^{2}\right)$$ (2)

For the bird predator:

$$\begin{array}{l} \Delta S^{2}=\;[{\left(e_{1}e_{2}\right)}^{2}{\left(\Delta Q_{4}-\Delta Q_{3}\right)}^{2}+{\left(e_{1}e_{3}\right)}^{2}{\left(\Delta Q_{4}-\Delta Q_{2}\right)}^{2}+{\left(e_{1}e_{4}\right)}^{2}{\left(\Delta Q_{3}-\Delta Q_{2}\right)}^{2}+\;\\ {\left(e_{2}e_{3}\right)}^{2}{\left(\Delta Q_{4}-\Delta Q_{1}\right)}^{2}+{\left(e_{2}e_{4}\right)}^{2}{\left(\Delta Q_{3}-\Delta Q_{1}\right)}^{2}+{\left(e_{3}e_{4}\right)}^{2}{\left(\Delta Q_{2}-\Delta Q_{1}\right)}^{2}]\\ /[{\left(e_{1}e_{2}e_{3}\right)}^{2}+{\left(e_{1}e_{2}e_{4}\right)}^{2}+{\left(e_{1}e_{3}e_{4}\right)}^{2}+{\left(e_{2}e_{3}e_{4}\right)}^{2}] \end{array}$$ (3)

where *e* is the estimate of receptor signal-to-noise and Δ*Q*_*i*_ is the difference in quantum catch between two spectra (crab and background) for a given receptor type. Noise is approximated by:

$$e_{i}\;=\;\omega_{i}/\sqrt{g_{i}}$$ (4)

where *ω*_*i*_ is the Weber fraction noise estimate and *g*_*i*_ is the relative abundance (or ratio) of the photoreceptor type. In the absence of species-specific information, *ω*_*i*_ was set at 0.05 which is appropriate for avian vision in high light situations [52] and also used in studies of fish visual systems [23, 53]. For each receptor type *g*_*i*_, the relative abundance of each was used [11, 19, 23, 26]. For the fish model, the ratio was set at 1:1 (*g*_*1*_ = *g*_*2*_) [11, 19, 23]. In birds, the relative abundance of their four photoreceptor types has been found to increase with sensitivity to longer wavelengths, but is highly variable both between species and eye region [26, 49]. For the bird model, *g*_*1*_:*g*_*2*_:*g*_*3*_:*g*_*4*_ was initially set as 1.5:1:1.5:2 [49, 50, 51] which represents the mean for the entire eye in *P*. *pacificus*. The UV component of avian vision is not modeled by our method, but this is unlikely to influence our results, as later discussed.

The units of chromatic contrast (Δ*S*) are Just Noticeable Differences (JNDs). Larger JNDs indicate greater color contrast to the visual system of the observer [11, 22, 23, 46]. When this parameter was less than 1, the crab was considered indistinguishable from its background. The literature suggests that values between 1 and 4 are difficult to distinguish, while JNDs greater than 4 would indicate that the crab is readily distinguishable [46, 53–55].

Additionally, achromatic contrast (or difference in luminance) can be used to identify targets on small spatial scales [17, 19–23, 51]. This is accomplished through paired or double cones, while chromatic contrast is processed from input of single cone cells. We calculated achromatic contrast for all crabs, under both fish and bird predators, as:

$$\left(\Delta S_{AC}\right)\;=\;\left(\Delta Q\right)/e$$ (5)

where Δ*Q* is the difference in quantum catch for crab and algae to the longest wavelength cone, and *e* calculated as in Eq 4.

#### Light Environment and Attenuation

Light field and water optical property data were generated in Hydrolight 5.2 (Sequoia Scientific) radiative transfer software from user specified inputs. As visual systems are sensitive to photons and not energy, irradiances were converted to quanta. The optical properties of the water column were determined from the New Case 1 Model using satellite derived Chlorophyll *a* concentration values (NASA GES DISC, Giovanni Web Service) and should be representative of clear Sargasso Sea water. A second case was also considered with high light attenuation comparable to conditions found in the Florida Keys coastal region, using previous local measurements of diffuse attenuation *K*_*d*_ (λ) [56].

Attenuation through the water column at each wavelength was modeled [6, 56, 57] from Beer's Law:

$$E_{d}\left(z\right)=\;E_{d}\left(0^{-}\right)e^{-K_{d}z}$$ (6)

where *E*_*d*_(z) and *E*_*d*_(0^-^) are downward irradiance at depth *z* and just below the surface and *K*_*d*_ is the downward diffuse attenuation coefficient (with (λ) notation dropped for clarity). From this, a transmission factor *T*(λ) was calculated [6, 26, 56, 57] for use in predator visual modeling:

$$T\left(\lambda \right)=\frac{E_{d}\left(z\right)}{E_{d}\left(0^{-}\right)}=\;e^{-K_{d}z}$$ (7)

Several of the above parameters (illumination, light transmission through the water column, and bird photoreceptor ratio) were varied to determine the impact of these factors on chromatic contrast. For all individual crabs, the impact of modifying model parameters was assessed by change in chromatic contrast, dividing the new chromatic contrast value by the initial values:

$$\text{Change}\;\Delta S\;=\;\frac{\Delta S_{mod}}{\Delta S_{ini}}$$ (8)

## Results

First, we describe observations of floating algal mats and *Sargassum* crabs encountered in the Sargasso Sea. The reflectances obtained from hyperspectral imagery of crabs and algae taken in the field are presented and compared. Then, we develop a conceptual modeling framework from our field observations. Using the conceptual and reflectance data, color matching by crabs is assessed through two different visual models: a dichromat fish and tetrachromat bird.

### 3.1 Environment and Organism Description

**Fig 1:**
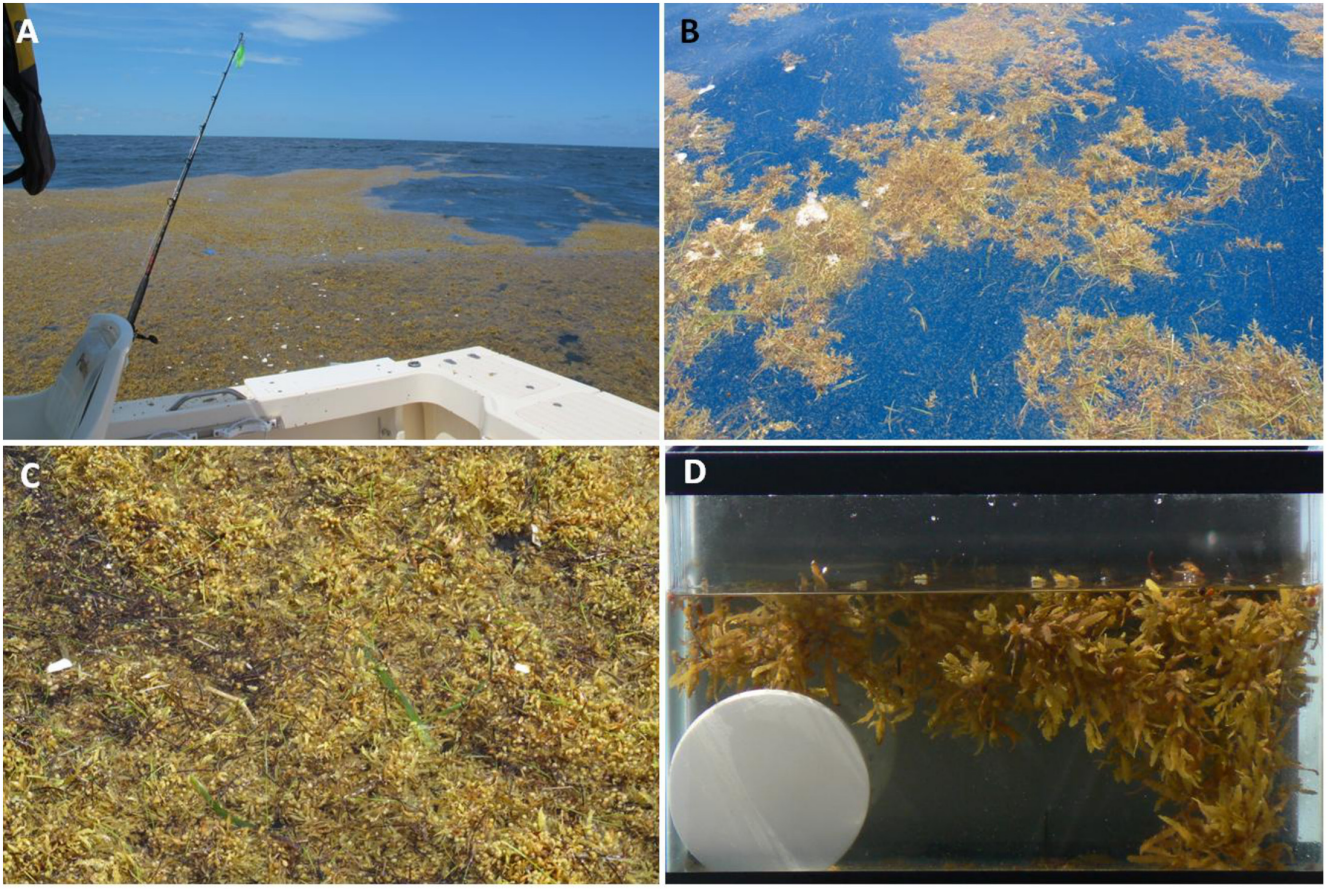
Photos of the floating macroalgae *Sargassum* encountered during this study in the Sargasso Sea, south of Bermuda. Individual aggregations can be extensive and densely packed (**A**) or more diffuse (**B**). Densely packed mats can be solid and partially emergent from the water column (**C**). The algae may float at several different depths and extend downwards from the surface by tens of centimeters, as depicted with a sample in a small tank (**D**).

Drifting mats of *Sargassum* were encountered sporadically throughout both cruises. *Sargassum* was observed in different densities and configurations, including expansive amorphous mats (Fig 1A), thin windrows, and isolated clumps of one or a few individual strands or “plants” (Fig 1B). Mats extended to different depths depending on the mean buoyancy of individual strands and the density of strands in a given mat. A “dense” mat was approximately 30 cm thick, though this was variable. Our observations revealed clumps penetrating to approximately 5 m below the sea surface. The upper surface of thick mats was often uniform and emergent (Fig 1C). The bottoms of the mats were more rugose, leading to a complex 3-dimensional environment (Fig 1D). For a camouflaging animal, this would allow for a multitude of depths and orientations in relation to the ambient light field.

**Fig 2:**
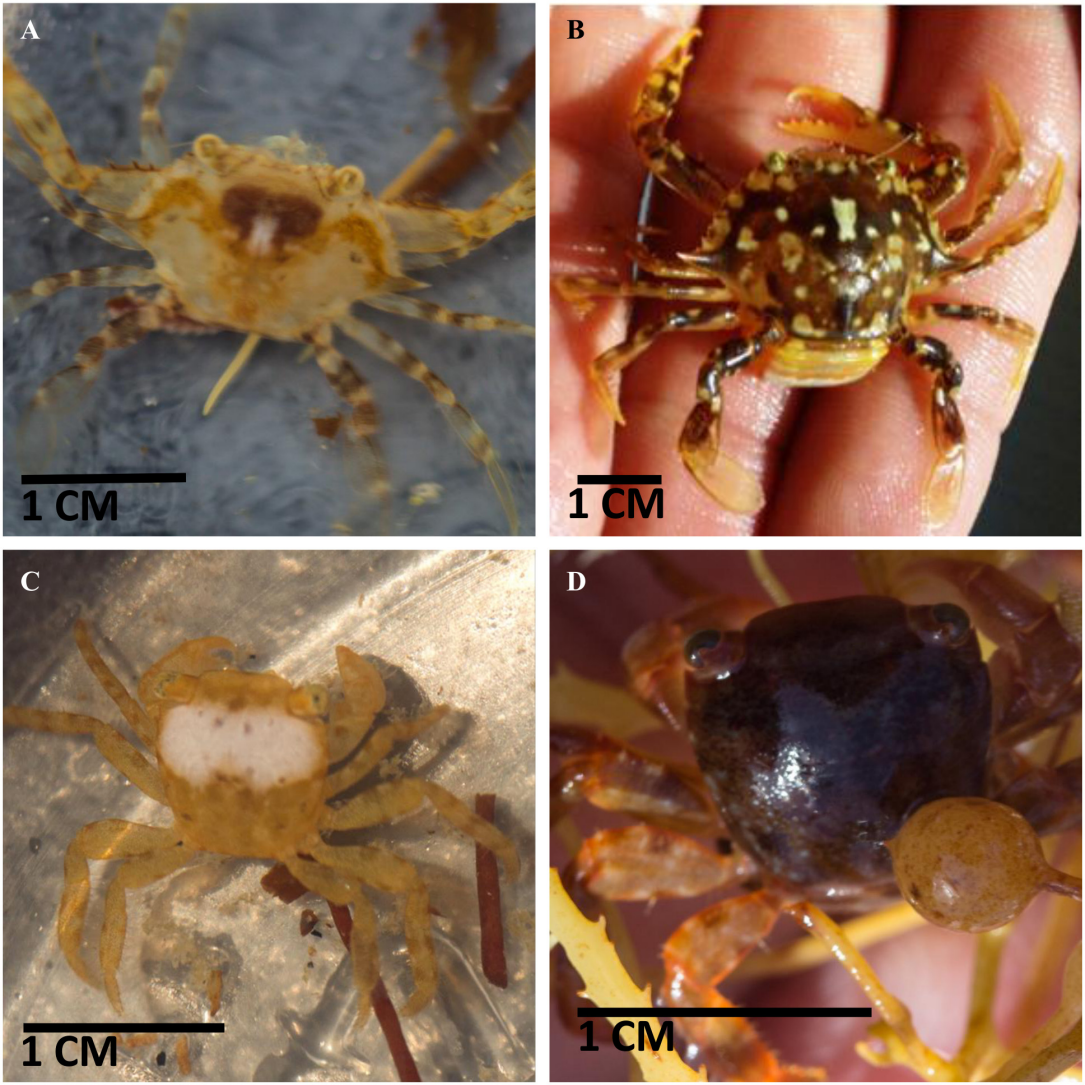
Photos of representative *Sargassum* crabs from two different species encountered in the Sargasso Sea. *Portunus sayi* display considerable variation in pattern from more uniform and pale (**A**, small adult male) to highly mottled (**B**, large adult female). Some aspects of patterning, such as the central dorsal white spot and m-shaped saddle marking, appeared in all adult individuals. The smaller species *Planes minutus* tended to have a more uniform coloration across the carapace (**C**, adult female). Some individuals had one or more white patches on the dorsal surface which ranged from small to covering the entire carapace and appendages. Several dark red *P*. *minutus* (**D**, adult male) were found drifting on a plastic bucket.

Two species of crab were found in the mats and used as model organisms in this study (Fig 2). The *Sargassum* Swimming Crab *Portunus sayi (Gibbes 1850)* was the larger of the two and had a yellow/brown, mottled appearance on its dorsal carapace. Of the 8 individuals (4 male, 4 female) encountered, the carapace width ranged from 0.50 cm to 2.53 cm and averaged 1.04 cm. Subjectively, the background shade of the carapace varied from yellow to dark brown and the mottled patterns varied greatly between individuals. Small *P*. *sayi* were lighter yellow overall, with dark markings. All individuals possessed a dorsal saddle mark with a central white patch. The patch covered approximately 4% of the carapace (Fig 2A and 2B). Larger individuals possessed additional small white spots. Limbs were generally marked with light and dark stripes perpendicular to the appendage. The carapace of the largest individual (Fig 2B) was darker overall, but retained the saddle marking. We have observed the same trend of darkening with size in other regions (Greater Florida Bay, Gulf of Mexico).

The Gulf Weed or Columbus Crab *Planes minutus* (Linnaeus 1758) was generally yellow in color (Fig 2C), ranging to light orange for some individuals. Twenty three individuals were encountered on *Sargassum* (12 male, 11 female), and carapace widths ranged from 0.31 cm to 0.80 cm, with a mean of 0.53 cm. Conspicuous white spots were apparent on 8 *P*. *minutus*, ranging in size from ~1 mm^2^ to the entire surface of the carapace. These were irregularly shaped, with 1–2 markings per individual. With the exception of these markings, individual *P*. *minutus* were generally uniform in color.

*P*. *minutus* were also found on a floating, red plastic bucket during the first cruise. These crabs were much darker in coloration than those in *Sargassum* (Fig 2D) and were treated as a separate subpopulation in this study. In the plastic bucket, 7 individuals (3 male, 4 female) ranged from 0.51 cm to 0.88 cm, with a mean of 0.69 cm. Six of these were dark red, with one possessing a large white spot covering approximately 75% of the carapace. One individual was brown in color, though darker than those found on algae. No significant difference in carapace width was observed between male and female for either species. For individuals collected on *Sargassum*, all crab colors were visually within those of algae samples. Ventral sides were pale yellow to white for both species.

**Fig 3:**
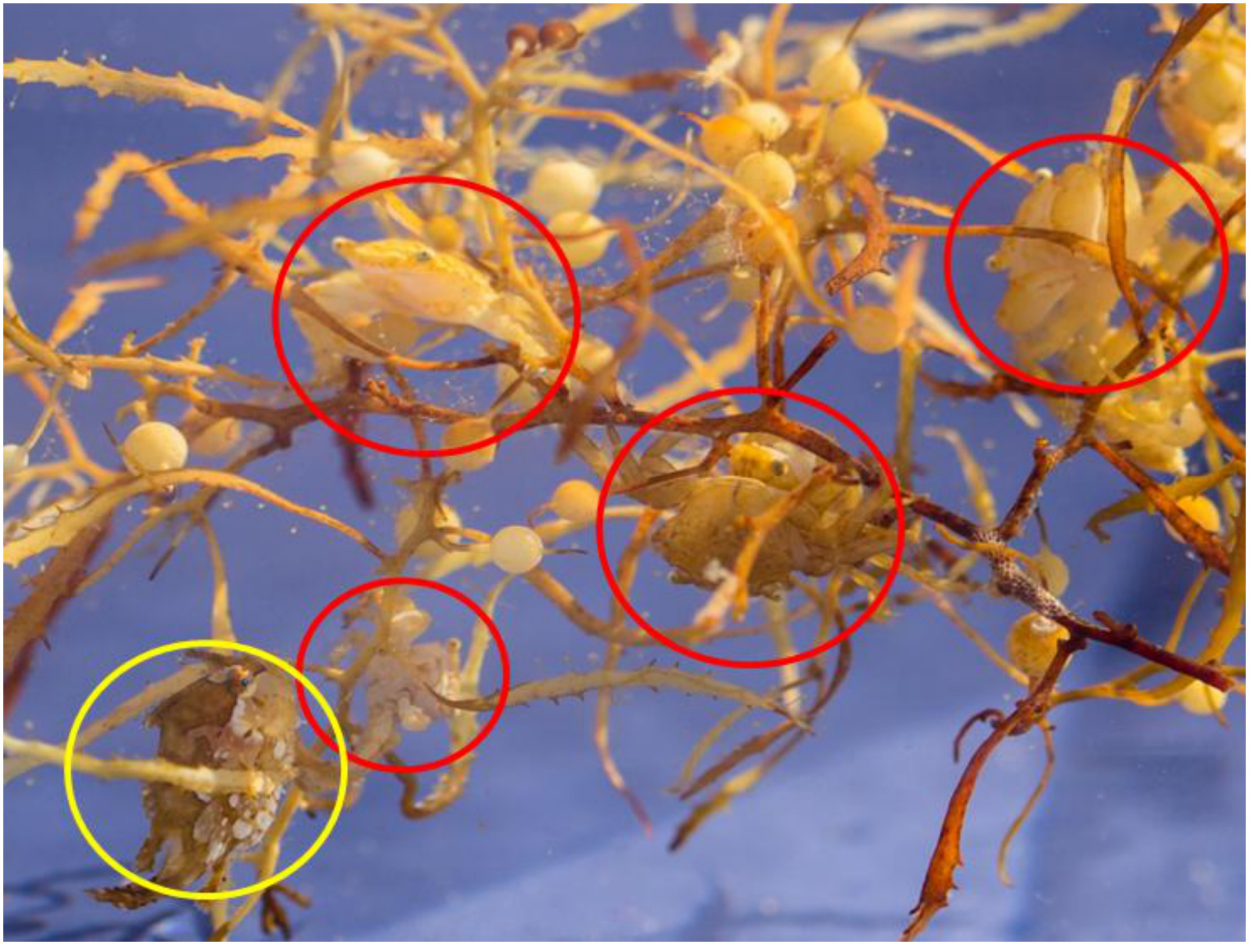
*Sargassum* crabs on algae in a small tank. Individual crabs (circled in red) did not show any preferred orientations relative to algae during short-term measurements in shipboard aquaria. Note the presence of a small frog fish, *Histrio histrio*, in the lower left (yellow circle).

The location and orientation of the crabs within the floating *Sargassum* mats were not directly observable with our collection methods. However, short term observations were made on deck in small tanks, where crabs were allowed to move freely within clumps of algae. Individuals were oriented in multiple directions clinging to algae fronds and stipes, with no clear trends in positioning or movement evident (Fig 3) for either species. Both species of crab appear to display counter-shading, though neither seemed to continually orient themselves to utilize this camouflage strategy. We have also observed adult *P*. *sayi* sitting emergent from the water column on top of mats in the Florida Keys, though this was not seen during the present study. While specific animal frequency data was not collected, multiple individuals were often encountered in the same dip sample, periodically in close proximity. Both species were encountered in the same sample on at least one occasion.

Water was clear without considerable amounts of phytoplankton or other light absorbing or scattering constituents. The mean Chlorophyll *a* concentration (Chl) in this region during the study period was estimated from the MODIS Aqua ocean color satellite to be ~0.1 mg m^-3^. Water clarity in this region, although highest in the summer (Chl = 0.05 mg m^-3^), was considered high throughout the year. During the study period, daylength was approximately 14 hrs with mostly clear sky conditions.

### 3.2 Reflectance of Organism and Background

**Fig 4:**
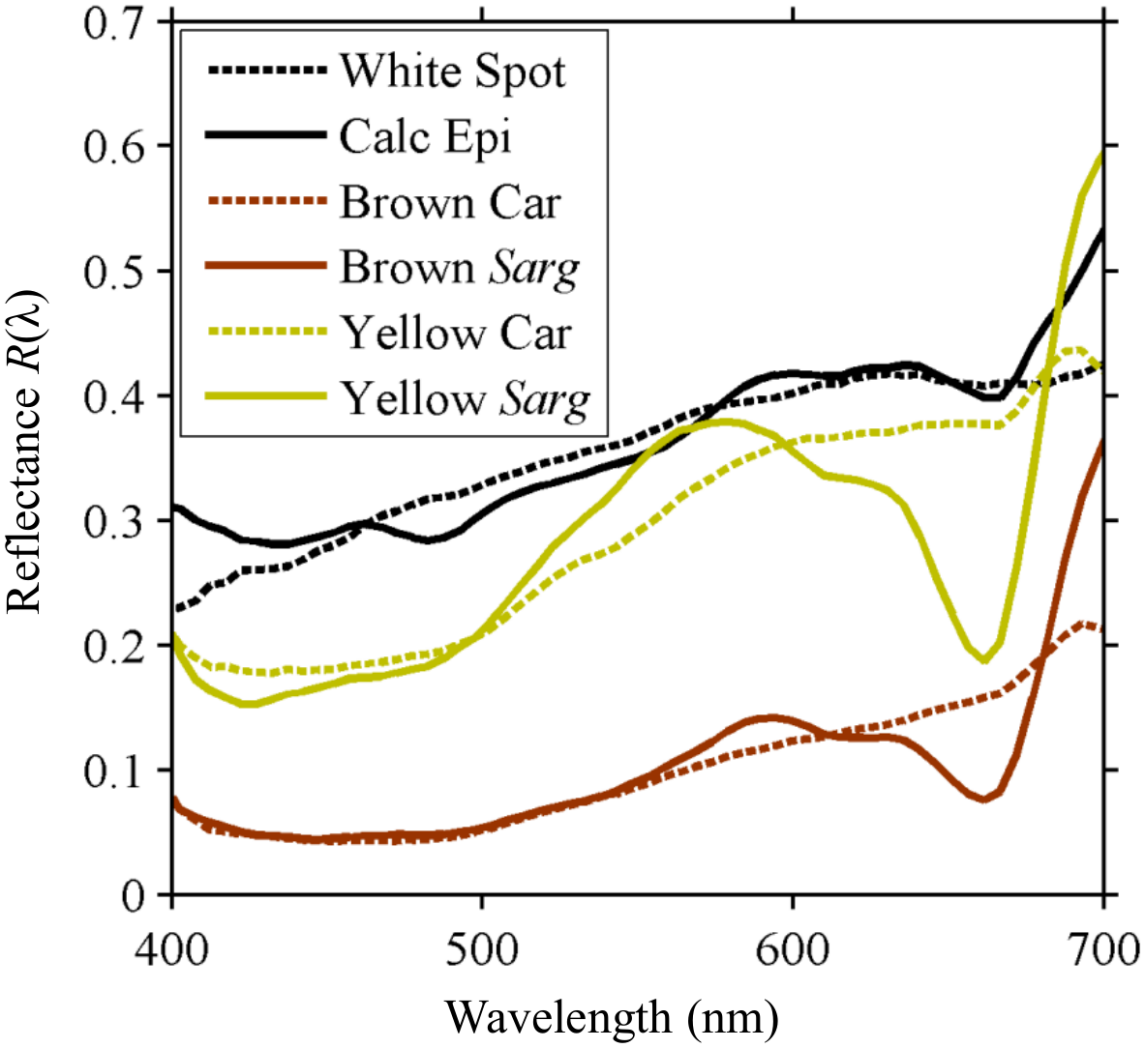
Reflectance of *P*. *sayi* pattern elements and subjectively corresponding habitat features. Reflectance *R*(λ) of the white, brown, and yellow areas on a randomly selected *P*. *sayi* carapace very closely matched the reflectance of calcareous epibionts, senescent brown, and healthy yellow *Sargassum*. Spectra of yellow and white areas on *P*. *minutus* showed similar correspondence. As with carapace and algae mean reflectance, maximum divergence appears in the far red.

**Fig 5:**
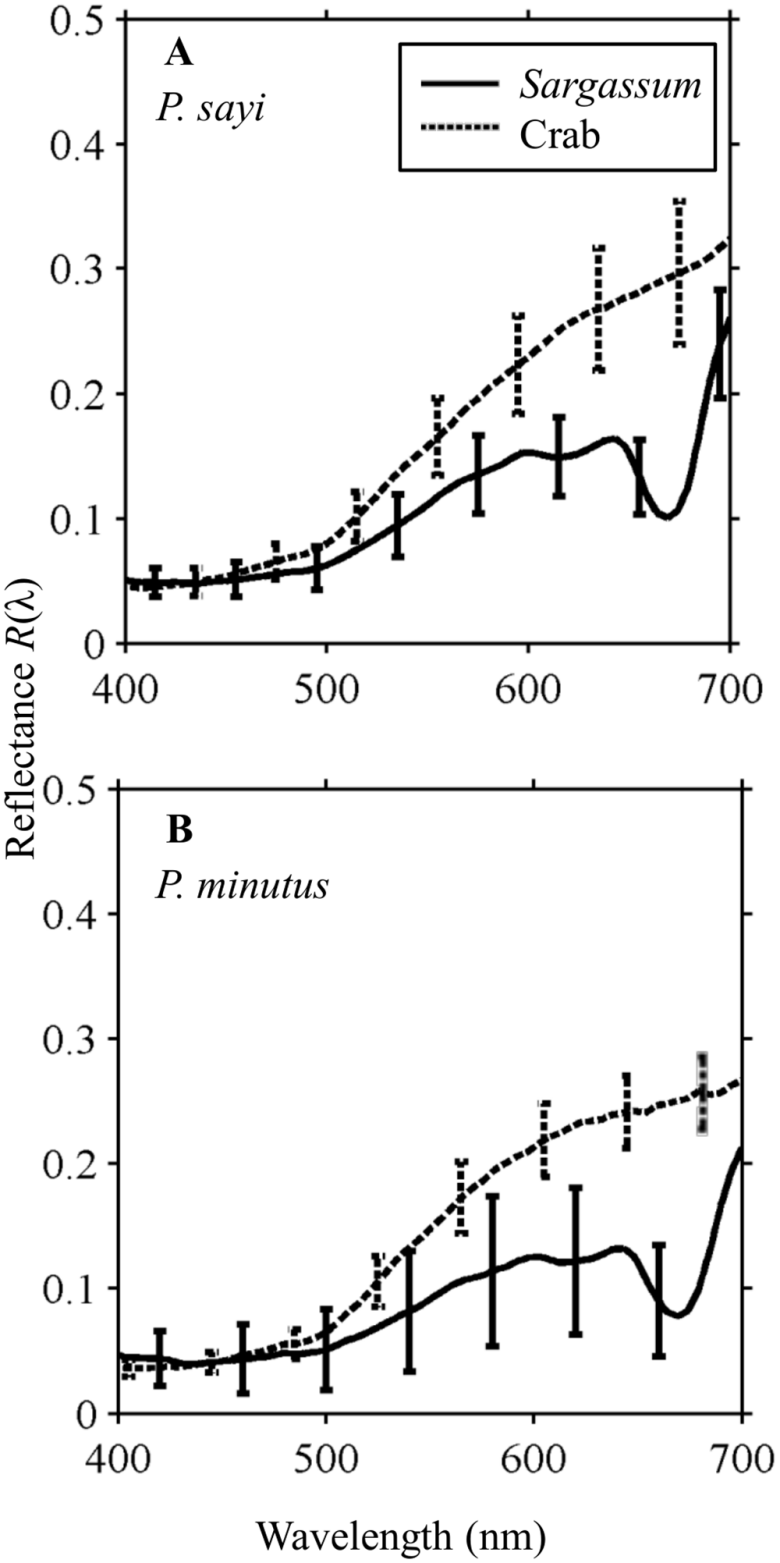
Mean and standard deviation of the measured reflectance spectra, *R*(λ), for a single randomly selected individual and its background obtained from a hyperspectral image of A) *P*. *sayi* and B) *P*. *minutus*. The greatest difference for both species appears around the secondary chlorophyll absorption dip (675 nm) present in *Sargassum* reflectance, but not observed for the crabs. Variation in *R*(λ) across the carapace is lowest in the blue and increases towards the red.

Hyperspectral imaging (HSI) was conducted on each individual crab and the associated background of *Sargassum* or red plastic. For an individual crab, the reflectance of the different color and pattern elements (yellow, brown, white) show good matching to elements of the *Sargassum* environment (Fig 4). Over the entire carapace and algae ROIs, both crab and algae showed low mean reflectance in blue wavelengths (400–500 nm), with values rising steadily into the orange and red (500–600 nm) (Fig 5A and 5B). Crab reflectance continued to gradually increase monotonically, while *Sargassum* flattened due to characteristic absorption features and then dipped sharply at the Chlorophyll *a* secondary absorption band (675 nm) before rising exponentially at the vegetative “red edge”. Standard deviation of *R*(λ) for both crabs and algae was lowest in short wavelengths, and increased into the red, representing the variability of *R*(λ) among all pixels in a ROI. *R*(λ) values were generally normally distributed for both species (S1 Fig).

**Fig 6:**
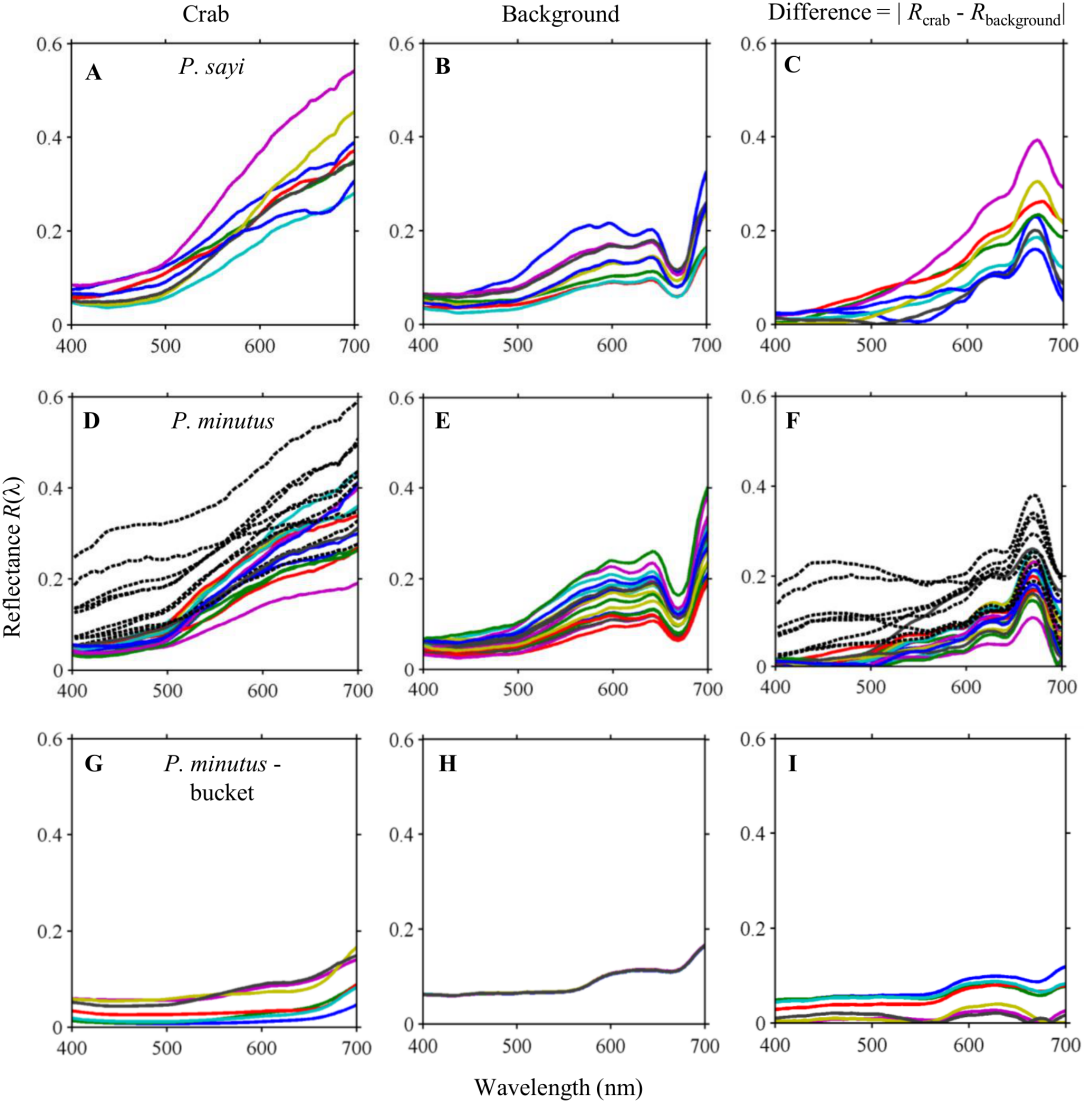
Reflectance *R(λ)* of individual crabs and associated backgrounds determined from hyperspectral imagery collected from the Sargasso Sea. Left panels show the reflectance spectra of individual crabs. Middle panels show spectra for the associated background (*Sargassum* or bucket). Right panels present the difference between the left (crab) and middle (background) panels. Error bars for each spectrum have been omitted for clarity, but were similar to that for the example individuals. Black dashed lines in panels (**D, F**) represent *P*. *minutus* with large white markings and higher reflectance than other crabs of both species. Individual *P*. *minutus* collected from a floating red bucket (**G**) all had substantially lower reflectance than crabs on natural algae. The greatest difference between crabs and algae was centered around the secondary absorption band of Chlorophyll *a* (675 nm) where a dip was found in the *Sargassum* reflectance spectrum.

Both *P*. *sayi* (Fig 6A, 6B and 6C) and *P*. *minutus* (Fig 6D, 6E and 6F) most closely matched the reflectance of their algal backgrounds in the blue, green, and yellow regions of the spectrum, with greater divergence appearing in the red. For all individuals of both species on algae, the largest difference was observed in a small region centered on the secondary chlorophyll absorption feature (Fig 6C and 6F) where the spectral differences were up to seven times greater than that at other wavelengths. For individual *P*. *minutus* with large white markings, enhanced reflectance was found in blue and green spectral regions (Fig 6D, black dotted lines) and resulted in abnormally high differences between crab and background in blue wavelengths (Fig 6F) as well as increasing the variation in *R*(λ) values for this species. Among the generally dark red *P*. *minutus*–bucket subgroup, reflectance was flatter and lower across all wavelengths than for the other groups (Fig 6G). The corresponding reflectance of the plastic was also extremely low (Fig 6H), and the greatest difference between crab and background reflectance in this group was observed between 575 and 675 nm (Fig 6I).

### 3.3 Discrimination of Crab and Background by Predators

**Fig 7:**
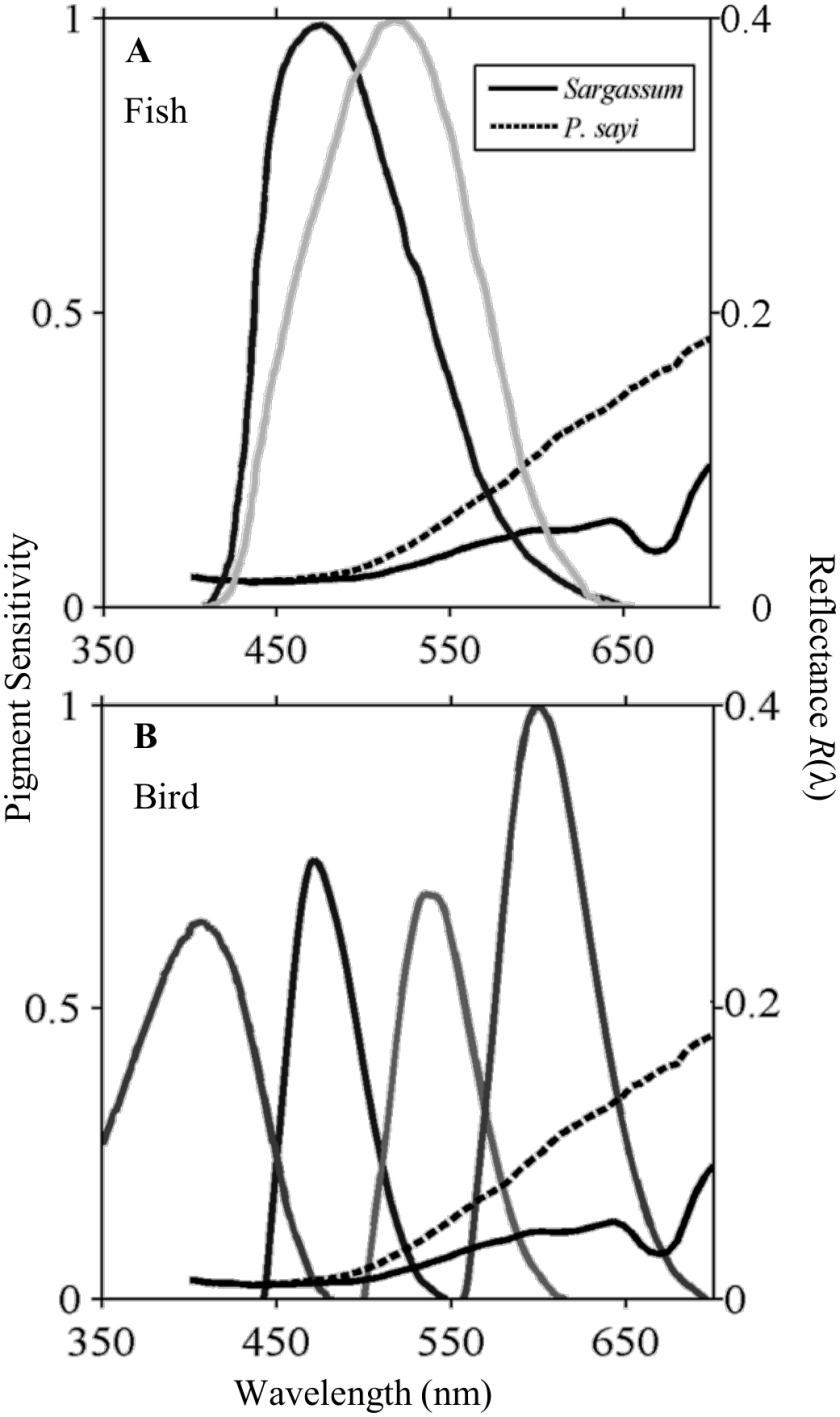
Normalized visual pigment sensitivities for dichromat fish mahi mahi (A), *Coryphaena hippurus* (Munz and McFarland 1979), and tetrachromat bird wedge-tailed shearwater (B), *Puffinus pacificus* (Hart 2004). *C*. *hippurus* is most sensitive to light in the blue and green wavelengths, while *P*. *pacificus* is also sensitive to red wavelengths. Sensitivity spectra (limited to 400–700 nm) are superimposed on sample *R(λ)* for crab and algae.

Following our observations of the environment and organisms (Sec. 3.1) and reflectance measurements of algae and crabs (Sec. 3.2), a conceptual framework was developed for assessing the color matching abilities of *Sargasum* crabs in the view of fish and bird predators (Fig 7). This framework allowed us to vary several of the parameters in the chromatic contrast model to emulate realistic environmental variation. Several “worst case” scenarios are presented for testing configurations of airborne and in-water predators, light field, and water column.

Two initial scenarios were considered based on predator type. Within the conceptual framework, the crab was placed on the background and observed with the dorsal surface facing orthogonal to the predator. *Sargassum* was treated as a planar surface comprised of the fronds of algae and opaque to light. For the fish predator, the algal surface was placed vertically. This is consistent with the strategy of *C*. *hippurus*, which attacks prey parallel to the water surface [58]. Depth was arbitrarily set at 15 cm, within a reasonable depth range for floating *Sargassum*. The illumination spectrum *I*(λ) was set as downward planar irradiance *E*_*d*_(λ) at 15 cm below the sea surface (S2 Fig).

In the bird predation scenario, the algal surface was placed horizontally at the surface of the water column with the crab emergent and observer in air, looking downward. The illumination *I*(λ) was set as downward planar irradiance incident upon the sea surface *E*_*d*_(0^+^) (S2 Fig). For both scenarios, *T*(λ) was initially set at 1 for all wavelengths.

**Fig 8:**
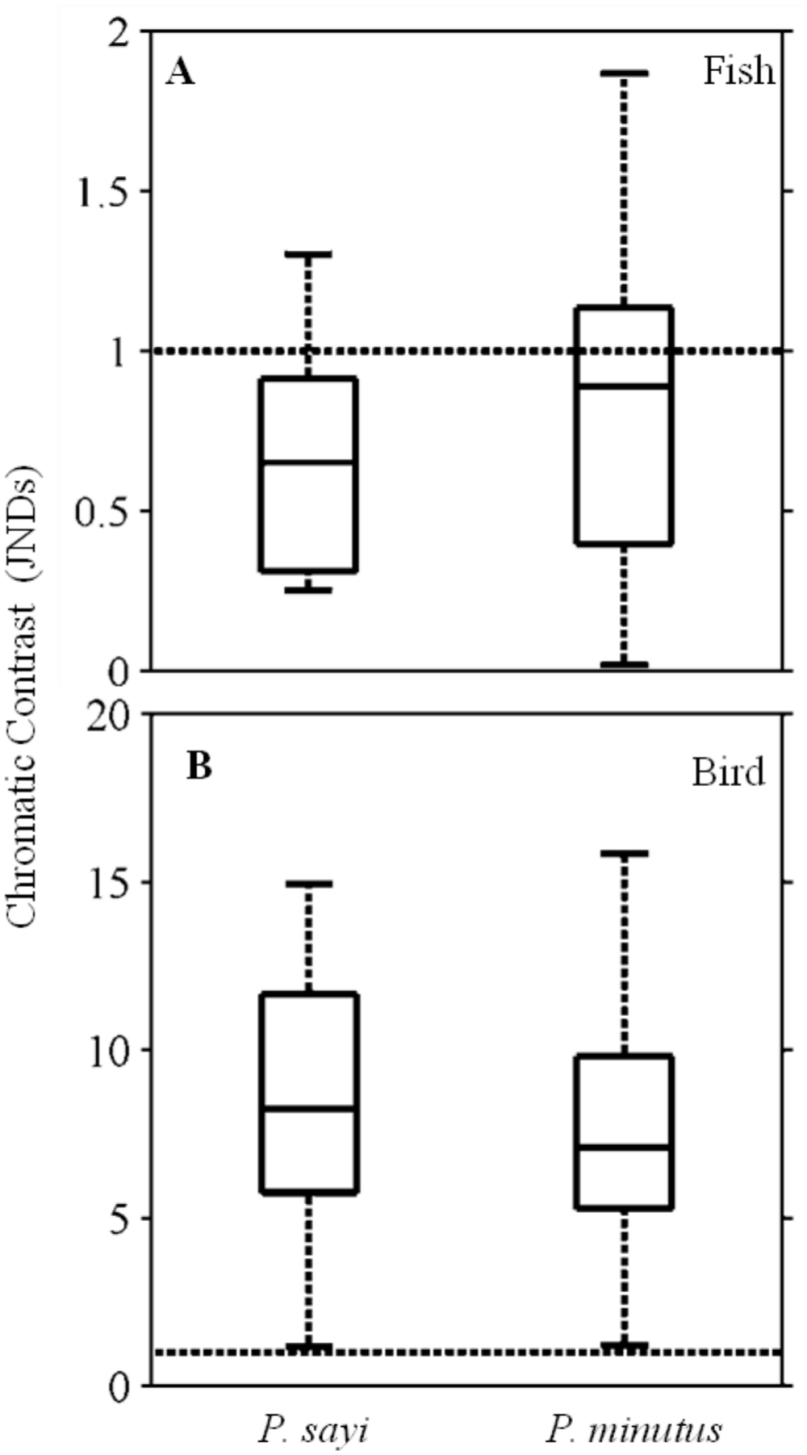
Crab chromatic contrast (Δ*S*) for initial model conditions when viewed by fish (A) and bird (B) predator models. Units are Just Noticeable Differences (JNDs), and values less than 1 (dashed line) indicate that the organism is not distinguishable from its background. Contrasts for the fish model were mostly below 1, while values for the bird were significantly higher.

**Fig 9:**
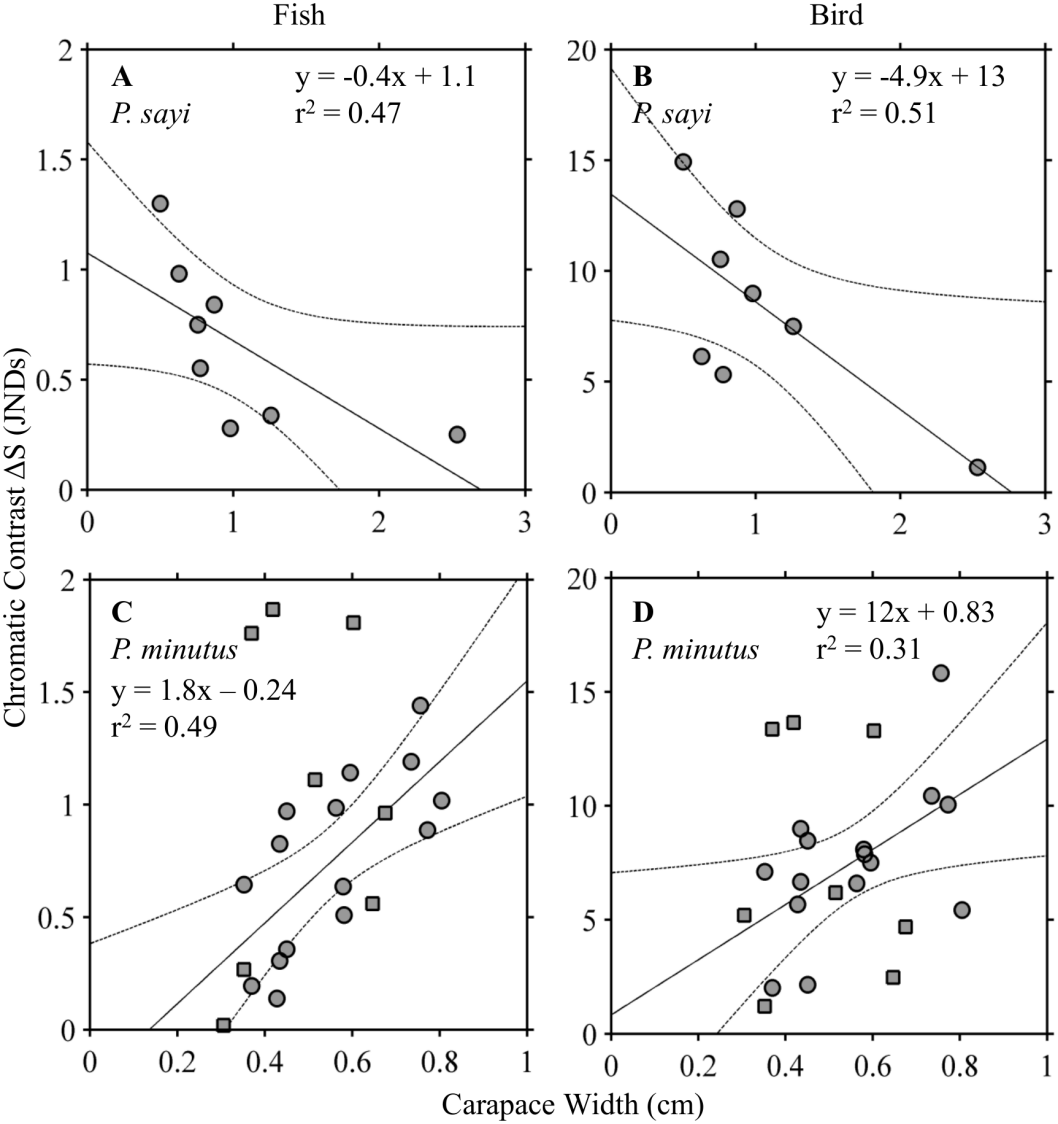
Chromatic contrast (Δ*S*) plotted against carapace width (CW) for each species under fish (left panels) and bird (right panels) visual models. A linear regression model was fit to each group. Δ*S* showed a strong, significant (p = 0.05) negative correlation to CW for both models in *P*. *sayi* (**A**, **B**). For *P*.*minutus* (**C**, **D**), significant positive correlation to CW was observed for individuals without large white patches (circles). This correlation was not observed for individuals with white patches (squares), which have been superimposed on the figure and were not used in calculating the regression. Dotted lines represent 95% confidence interval.

The parameter chromatic contrast, in units of Just Noticeable Differences (JNDs), quantified how discernible each crab was from its background given the visual capabilities of the predator and the chosen environmental scenario. On *Sargassum*, *P*. *sayi* and *P*. *minutus* achieved approximately equal contrast under initial conditions (Fig 8). The dichromat piscine predator was not able to reliably discriminate between background and crab for either species, though some individual crabs showed JNDs above the threshold detection value of 1 (Fig 8A). The tetrachromat avian predator, by contrast, was able to distinguish between crab and background for both species with no individuals below the threshold (Fig 8B). Mean contrasts were not significantly different (p = 0.05) between *P*. *sayi* and *P*. *minutus*. Within each group, individual crabs achieved a wide range of chromatic contrast with their background. For *P*. *sayi*, contrast was inversely correlated to carapace width for both dichromat fish and tetrachromat bird predators (Fig 9A and 9B), while *P*. *minutus* showed positive correlation (Fig 9C and 9D).

**Fig 10:**
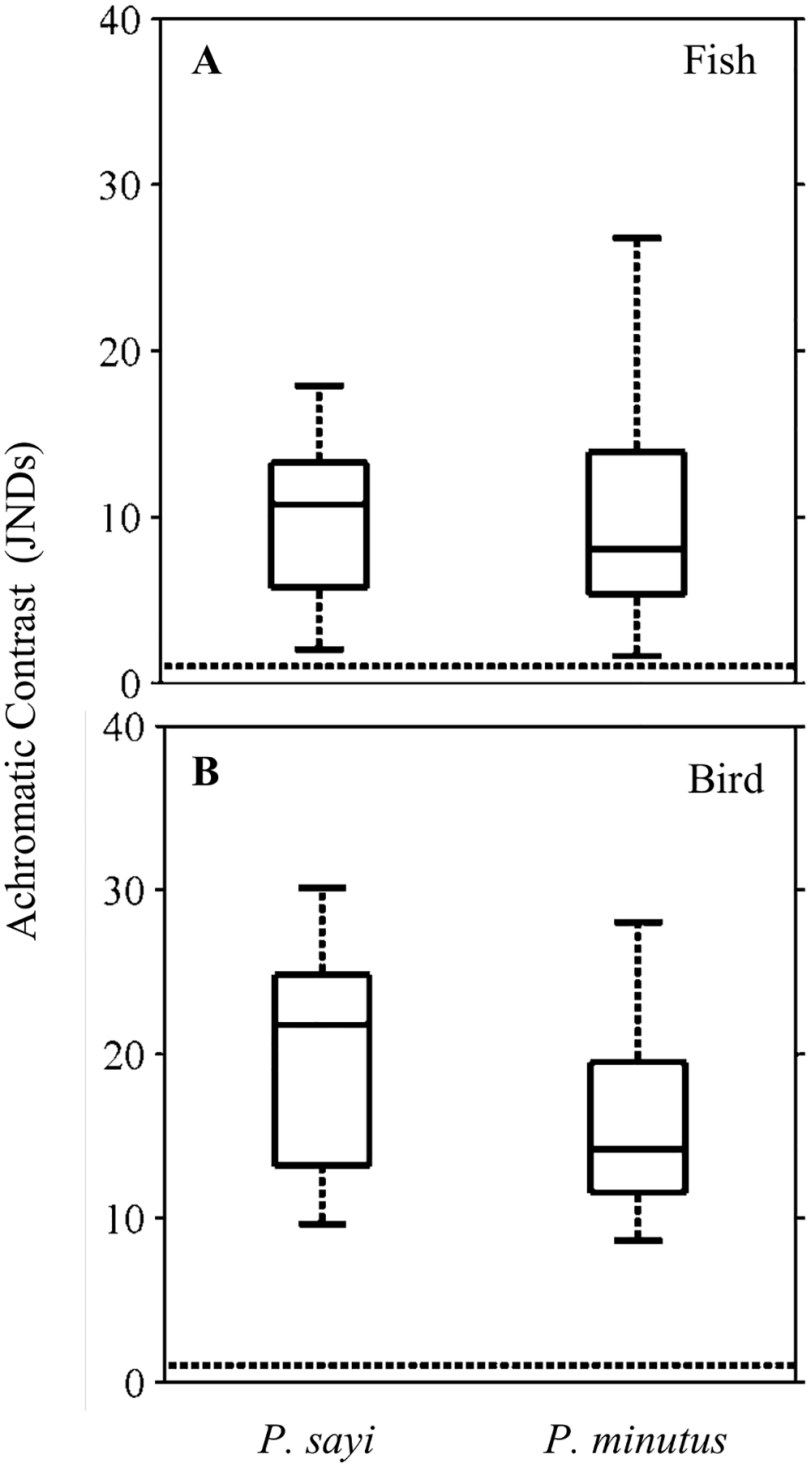
Achromatic contrast (Δ*S*_*AC*_) for initial model conditions when viewed by fish (A) and bird (B) predator models. Units are Just Noticeable Differences (JNDs), and values less than 1 (dashed line) indicate that the organism is not distinguishable from its background. Achromatic contrast for both crab species were not significantly different (p = 0.05) for either predator model. All individuals were above the threshold value of 1 for both predators.

Achromatic contrast was an order of magnitude higher than chromatic contrast for the piscine model (Fig 10A) for both *P*. *sayi* and *P*. *minutus*, and approximately twice as high for most individuals as chromatic contrast for the avian predator (Fig 10B). Contrasts between crab species were not significantly different (p = 0.05). No contrast trends with body size were observed.

### 3.4 Discrimination of Crab and Background—Model Variation

#### Avian Photoreceptor Ratio

**Fig 11:**
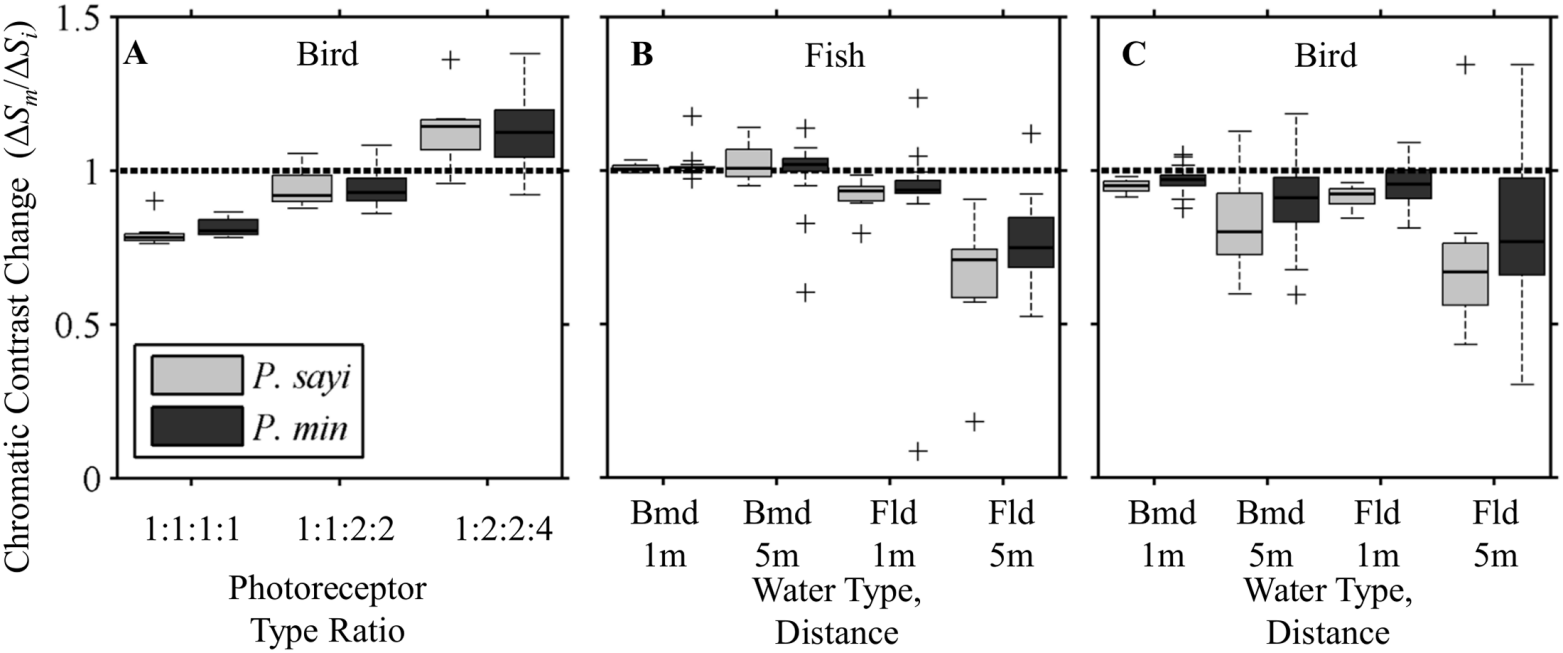
Impact of bird model photoreceptor ratios and water column attenuation on chromatic contrast (Δ*S*). Bars represent median contrast for each crab group relative to Δ*S* for the initial condition. Dashed line represents a ratio of 1 (no change). For bird photoreceptor ratios (**A**), the unity ratio achieved the lowest chromatic contrast. Contrast increased with increasing proportion of red-sensitive photoreceptor types. Chromatic contrast at 1 and 5 m distance in a low (Bermuda/Bmd) and high (Florida/Fld) attenuation water columns were compared to null attenuation (T = 1, Eq 1). Simulating attenuation of light with distance generally decreased contrast values for both fish (**B**) and bird (**C**) predators, though this was highly variable on an individual basis with some individuals becoming more distinguishable (> 1). Outliers (+) were observed for both crab species. Decrease in chromatic contrast was most pronounced for the highest attenuation condition (Fld, 5 m).

For the avian predator model, multiple photoreceptor ratios were considered. Initially, the mean ratio of 1.5:1:1.5:2 was used. A unity ratio 1:1:1:1 and two ratios found in birds were examined: 1:1:2:2 and 1:2:2:4 [26, 49]. Due to the presence of outliers, median chromatic contrast for each group was used in comparing the effects of receptor ratio. Median JND increased for all crab populations, as well as most individuals, with increasing relative numbers of long wavelength receptor types. *P*. *sayi* group median chromatic contrast was 22% lower than initially at 1:1:1:1, 8% lower at 1:1:2:2, and 14% higher at 1:2:2:4. For *P*. *minutus*, the corresponding changes were 20% lower, 7% lower, and 12% higher for the respective ratios (Fig 11A). Several individuals in both species showed decreased contrast (< 1) at the 1:2:2:4 ratio. These crabs had relatively low reflectance in the blue relative to most individuals.

#### Attenuation through Water Column

**Fig 12:**
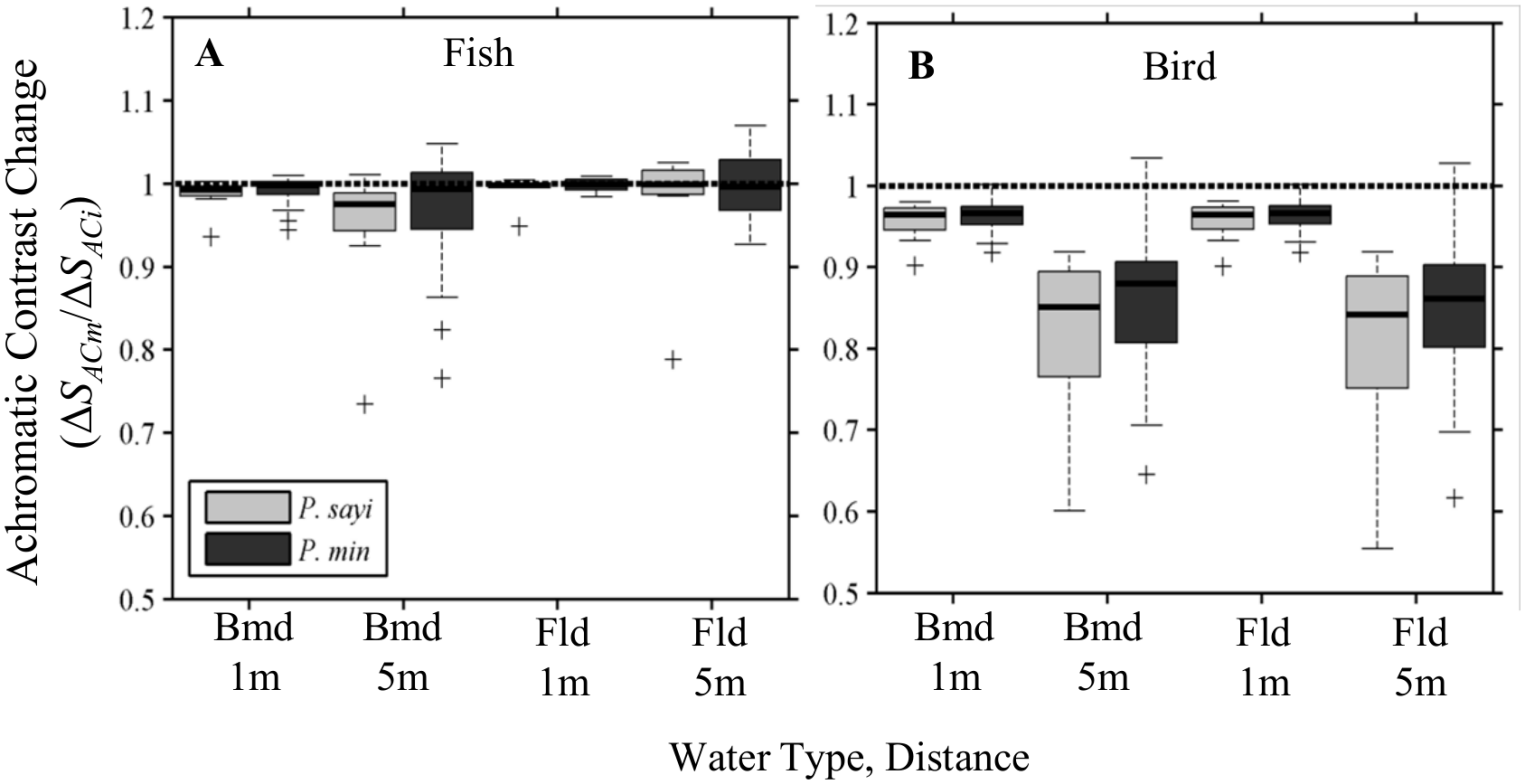
Impact of water column attenuation on achromatic contrast (Δ*S*_*AC*_) to fish (A) and bird (B) models. Bars represent median contrast for each crab group relative to Δ*S*_*AC*_ for the initial condition. Dashed line represents a ratio of 1 (no change). Decrease in achromatic contrast was generally low, except for the avian predator under the 5 m conditions, where median Δ*S*_*AC*_ of both species decreased by 15% and up to 50% for some individuals. Outliers (+) were observed for both crab species.

Attenuation by water with observer distance was investigated by varying *T*(λ). For the fish, *T*(λ) was calculated using horizontal distance through the water column in place of depth *z* (Eq 7). When evaluating attenuation by water for the bird, the predator was considered to have its head underwater during active foraging, and transmission across the air-water interface was not considered. An overlaying water column of varying depth was added to the initial scenario. Illumination *I*(λ) as *E*_*d*_(λ) was generated for each depth, and *T*(λ) calculated. Attenuation was estimated for two water types with differing optical characteristics, corresponding to the Bermuda Sargasso Sea and coastal Florida (S3 Fig) where we have also encountered *P*. *sayi*. Attenuation by the water column with increasing distance between target and observer decreased discriminability of crabs and algae for both predators. Low sensitivity was observed for the piscine predator (Fig 11B), which showed negligible (< 10%) decrease in chromatic contrast under all but the most attenuating Florida condition at 5 m depth, with reductions in median contrast of 29% for *P*. *sayi*, and 25% for *P*. *minutus*. The impact of attenuation on chromatic contrast was somewhat more pronounced for the bird predator model at intermediate attenuation than for the fish (Fig 11C). Under the most attenuating 5m Florida condition, reductions in median contrast were 33% for *P*. *sayi*, and 23% for *P*. *minutus*. Some individuals showed an increase in contrast with attenuation. This was restricted to those *P*. *minutus* with large, dorsal white spots and a single *P*. *sayi*. The effect of attenuation on achromatic contrast was negligible for the fish predator, and somewhat more pronounced for the bird (Fig 12) with median reductions under 5% at 1 m for both water types, and of 16% (Bmd) and 15% (Fld) for *P*. *sayi* at 5 m, and 14% (Bmd) and 12% (Fld) for *P*. *minutus*. Physically, for both water types the discrepancy in reflected signals between crab and algae diminished with increasing absorption and scattering with distance through the water column, particularly in the highly absorbed red wavelengths (S4 Fig).

#### Illumination and Visual Scene Reflectance

When illumination spectra *I*(λ) was altered from solar noon (initial scenario) to a red-shifted low solar angle (1 hour before sunset), a < 1% increase from the initial conditions in median contrast for each group of crab was observed for the bird visual model under sunset illumination, with no change in contrast evident for the fish at the given precision (1 decimal place). Varying *R* ^*b*^(λ) as a linear mix of algae and water had no discernible impact on chromatic contrast at the level of precision used for either predator.

## Discussion

We hypothesize that camouflage in the *Sargassum* ecosystem will be well developed due to its location at the air-water interface, the total lack of hard-cover retreats from predation, and the presence of multiple classes of visual systems. Indeed, we found that the spectra reflected from the crabs were very close to the background *Sargassum* (or red bucket), except at far red wavelengths. Important differences were found between the two species in terms of size when viewed by piscine and airborne predators.

### Discrimination by Predators Relative to Body Size

For *P*. *sayi*, chromatic contrast showed a strong decrease with increasing carapace width. In other words, the larger crabs were able to better match their background color than the smaller crabs. The sample size (n = 8) was relatively small given the variation in *R*(λ) for this species. Even though there was only a single very large *P*. *sayi* encountered in this study (2.5 cm wide), the correlation remained significant without this individual for body sizes from 0.5 to 1.3 cm (p < 0.01). *P*. *sayi* is known to change color on short (several hours) time scales, which may be employed in dynamic camouflage [59]. Expansion and contraction of chromatophores beneath the transparent carapace changes the color of the animal, and may provide a better match to its general surroundings [9, 60, 61]. Apparently, the background matching ability of *P*. *sayi* improves with size and/or age. The smaller crab, *P*. *minutus*, however, showed an opposite trend with body size, such that the larger individuals showed a greater contrast to their background than the smaller ones. Unlike *P*. *sayi*, coloration in *P*. *minutus* is not known to be dynamic and only changes over long time scales [5, 9]. As discussed further below, the individuals found on the dark red bucket had a significantly darker red color than those found on *Sargassum*, indicating an ability to acclimate to differently colored backgrounds. Color matching increased with carapace width in *P*. *sayi* and decreased in *P*. *minutus*. These results suggest that differences in body size, patterning, and camouflage strategy may be significant between these species [62]. Further research on color change in *P*. *sayi* and pattern heterogeneity in both species, determined from hyperspectral images, is forthcoming.

### *Planes minutus* on Floating Debris

By a stroke of coincidence, the present work sheds light on a much earlier study. The *P*. *minutus* encountered on a drifting bucket were of a deep red color not found in the natural algal population, which mirrors an observation by Crozier [5] of “mahogany” *P*. *minutus* on a floating cedar log. Unable to induce color change in adult *P*. *minutus* by placing them on varying backgrounds in a series of experiments, he postulated that this species may change coloration to match substrate over long periods. Hitchcock [9] described the crabs’ chromatophore responses, but found no clear indication that the species attempted to match new backgrounds over several days. In the present study, bio-fouling on the bucket indicates a significant period adrift. A prolonged duration within the bucket is supported by the significantly (p = 0.02) larger size of the bucket *P*. *minutus* as opposed to those from *Sargassum*. *P*. *minutus* is evidently capable of morphological color change on long time scales, possibly related to pigment deposition during molting [10, 61, 63], in contrast to the more rapid changes of *P*. *sayi* [59]. The present observation confirms Crozier’s initial discovery that *P*. *minutus* possesses an ability to match backgrounds with a wide color range, even beyond those encountered on evolutionary time scales.

### Predator Visual Systems

The ratio and spectral sensitivities of observer photoreceptor types can alter the chromatic contrast of a given target by an order of magnitude. *Sargassum* crabs match their algal background least closely in the red wavelengths. Model runs confirmed that the presence, as well as the relative abundance, of long wavelength sensitive photoreceptors increased chromatic discrimination between crab and background. While there is variation in cone cell sensitivity across bird taxonomic groups [49], measured or modeled data is still lacking for many bird species. This variation is greatest in shorter wavelengths, where birds possess either an ultraviolet (UVS) or significantly long-shifted violet sensitive (VS) type. Variation is considerably lower in longer wavelengths, as the spectral sensitivities of the medium, short, and long wave cone pigments (SWS MWS, and LWS, respectively) are relatively conserved [49]. The model bird in this study, *P*. *pacificus*, has the violet type. Work by Chiao et al. [23] has shown that color discrimination may not be greatly affected by small shifts in maximum absorption by visual pigments, and so the potential differences between the model system selected and actual avian predators is likely negligible for the purposes of this study. It must be noted that *P*. *pacificus*, like all known birds, is sensitive to wavelengths shorter than 400 nm. This is the lower bound of our visual model, due to the technical limitations of the hyperspectral imager. Discrepancies in *R*(λ) between crabs and algae in the ultraviolet would not be accounted for in our chromatic contrast calculations. However, inclusion of lower wavelengths would not significantly change our results. Crabs and *Sargassum* are very closely matched in *R*(λ) at 400nm, and our study assumes that this relationship persists in the UV, and Δ*Q*_*VS*_ would remain unchanged. The enhanced discrimination ability of birds in this system is apparently due to sensitivities in red wavelengths. Any divergence in *R*(λ) between crabs and background below 400 nm would only increase discrimination in the bird visual model, which is already clearly able to differentiate crab and algae by color.

In addition to chromatic contrast, birds, fish, and other animals are known to utilize achromatic contrast (or difference in luminance) to identify targets on small spatial scales. Many studies of animal camouflage have investigated this visual channel [17, 19, 20–23, 51, 54, 70, 73]. Our results indicate that the achromatic contrast between crabs and algae is up to an order of magnitude higher than the chromatic contrasts calculated in this study, and less affected by attenuation. The luminance channel may therefore be important for predators trying to detect crabs which are well matched to the algae in terms of chromatic contrast.

The visual systems of other important predators, like the Sargassum frogfish *Histrio histrio*, have yet to be studied and may be drivers of camouflage in this environment. *H*. *histrio* is a voracious ambush predator, and gut contents frequently contain *P*. *sayi*, *P*. *minutus*, and shrimp [64]. Visual sensitivity data is not available for *H*. *histrio*, but prey survival experiments on red, green, and yellow artificial *Sargassum* indicate that *H*. *histrio* may utilize color discrimination and camouflage breaking to detect prey [65]. The fish we modeled in this study, *C*. *hippurus*, is a widely distributed pelagic predator not endemic to the mats. Pelagic fish such as *C*. *hippurus* have visual pigments adapted to open-ocean ambient light fields [43, 48]. Visual pigments in fish adapted to *Sargassum* mats may be sensitive to longer wavelengths. Behavioral and molecular evidence for color discrimination by crabs, as well as the use of this ability in behavioral tasks like mate and habitat selection, is increasing [66–68]. Whether either *Sargassum* crab is able to utilize color information in guiding behavior is unknown, but seems possible in light of evidence for visual habitat discrimination by *Sargassum c*rustaceans [39, 69].

### Chromatic Modeling

Multiple models exist for estimating discrimination of color. The Vorobyev and Osorio model is widely used and supported by experimental data [4, 11, 16, 19, 21–23, 26, 46, 53, 54], and was considered appropriate for this study. Color constancy is here modeled by the von Kries transformation [21–23, 46, 54, 70], which incorporates both illumination *I*(λ) and the mean reflectance of a scene, *R* ^*b*^(λ). Varying the light field between noon and sunset conditions showed virtually no impact on the chromatic contrast of crabs for either predator. For this reason, there would likely be little difference in chromatic contrast between the horizontal downwelling irradiance used in the conceptual model and a more realistic sidewelling illumination. This has been observed [22] when measured downwelling and sidewelling irradiances were compared using the Vorobyev and Osorio model. Effects at the air-water interface may impact chromatic discrimination by birds beyond what has been considered here. While the red shifted light at sunset may slightly increase contrast for the bird, enhanced glint with low sun angle [71] would make target detection more difficult. Other surface effects like bubble production, spatial resolution, and signal loss through transmission across the air-water interface for submerged crabs would also make it more difficult for flying birds to detect prey.

A radiative transfer model was used to generate illumination data for both the surface and depth. Such sophisticated light modeling might not be necessary for deep, clear oceanic “type 1” water columns where simple estimates of illumination can be sufficient for most applications. However, more refined treatment of the spectral absorption and scattering properties of the water column can be important for assessing camouflage in many ecosystems. In optically shallow waters, for example, reflectance from the seafloor impacts the spectral shape, magnitude, and polarization characteristics of the light field [2]. Organisms that hide in such environments have to match downwelling light from the sun, as well as upwelling light reflected from the seafloor (sediment, seagrass, corals, etc.) and often match the polarization conditions that may be visible to certain predators [2].

Here, different spectral light attenuation properties were important to show the influence of water depth on background matching. The differences in reflected radiance between the crab and *Sargassum* became less pronounced with predator distance (S4 Fig). This is because water preferentially absorbs red photons [56]. Therefore, the reflected light from the crab and *Sargassum* in red wavelengths is absorbed by water molecules and primarily green photons (500–550 nm) reach the predator. This means that for fish swimming at the mats from a distance (e.g., *C*. *hippurus*), the crabs will be much harder to detect than for small fish endemic to the mats (*H*. *histrio*). Similarly, the crab was much less discernible at 5 m distance to a bird foraging at the sea surface in both Bermuda and particularly in the more turbid Florida waters.

The background reflectance, *R* ^*b*^(λ), used in this study was the average reflectance of a *Sargassum* sample imaged on deck, and is not fully representative of the true environment. A more accurate spectrum would possibly include dark water or glint, and require knowing the field of view of the predator. Simple linear mixing of algae and water spectra (Sec. 3.4), however, indicated that variation in *R* ^*b*^(λ) played a minimal role in overall chromatic contrast.

### Comparable Camouflage Systems

While direct comparison between studies is difficult, it appears that *Sargassum* crabs generally demonstrate similar or lower contrast to their natural background than multiple other cryptic species. In crabs, perhaps the most studied groups in regards to color, camouflage, and patterning are fiddler and shore crabs. For example, the fiddler crab *Uca vomeris*, when subject to predation by birds, achieves a similar color match to *Sargassum* crabs on a more static background (mud flat). When under low pressure from birds, these crabs showed significantly higher chromatic contrast (and therefore a poorer color match) compared to either high-predation *U*. *vomeris*, or the *Sargassum* crabs *P*. *sayi* and *P*. *minutus* [8]. However, a different fiddler crab, *U*. *tangeri*, has been shown to achieve much lower contrast (and better matching) against birds [21]. The shore crab *Carcinus maenas* has similar chromatic contrast to *Sargassum* crabs against natural backgrounds to an avian visual model [17]. In this study, *C*. *maenas* also showed a color change response to background albedo. Similarly, the horned ghost crab *Ocypode ceratophthalmus* exhibits both dynamic color change and a significant degree of background matching [16], though direct comparison to the present study is difficult as the authors did not quantify color matching in either tetrachromat or dichromat visual space. Like *P*. *Sayi* and *P*. *minutus*, the kelp crab *Pugettia producta* is also found closely associated with macroalgae. This crab exhibits an ontogenetic color shift: juveniles are found in red algae turf habitat and are generally dark red, and then migrate to off shore kelp beds and change to a brown or amber coloration [63] during adulthood. While we are unaware of any research on background color matching in this species, survivorship studies indicate that the distinct color morphs of *P*. *producta* are well camouflaged against macroalgae [10, 62]. In a comparable terrestrial paradigm, researchers found that crab spiders hiding on flowers had similar or higher chromatic contrast values against both trichromatic prey and tetrachromat avian predators than *Sargassum* crabs [72, 73]. Extensive research on cuttlefish has yielded a range of contrasts relative to background, but *Sargassum* crabs appear to achieve a comparable match to their background in the view of fish predators [19, 22, 23].

### Conclusions

*Sargassum* crabs were found to be extremely well matched to their background over most of the visible spectrum and were not distinguishable from algae in the view of a dichromat fish predator. Tetrachromat birds, by contrast, were able to discriminate between the color of *Sargassum* crabs and algae because of photoreceptors at far red wavelengths, corresponding to the chlorophyll absorption maximum. Interestingly, only one species of crab previously studied had better background matching to avian predators than the *Sargassum* crabs, but that species was found in mud flats without a noticeable chlorophyll absorption peak [21]. The ability to more closely match their algal background from visual detection by a fish compared to a bird potentially suggests that *Sargassum* crab camouflage may be driven more by predation by fish than birds.

Chromatic contract was considered under multiple environmental scenarios including diurnal variation in illumination, signal attenuation by water with distance, predator visual adaptation to patch size, and predator photoreceptor ratios. Photoreceptor ratio in avian predators and signal attenuation over distance had the largest effects on chromatic contrast. Attenuation of the light reflected from the crab and *Sargassum* generally decreased the chromatic contrast of crab and background with distance for both the fish and bird predators. With increasing distance in the real environment, factors such as veiling light, observer spatial resolutions, and occlusion of the target by algal fronds will also play a role in discrimination by predators [6, 26]. Additionally, some of the unique properties of air-water interface such as sun glint, internal reflection of photons and the presence of waves, bubbles and foam may also play a role that has yet to be considered [74, 75].

*Sargassum* crabs have convergently evolved coloration and patterning to match their background in an environment with no hard cover refuge and predation from both above and below the water surface. High resolution spectral measurements in the far red wavelengths (> 650 nm) were necessary to model chromatic contrast in this ecosystem. Hyperspectral imaging provided information on the variability associated with an individual carapace or algal sample that could not be effectively quantified using fiber optic spectrometry, and allowed for modeling the effects of variation in light field and visual system parameters on chromatic contrast. Forthcoming studies will utilize the spatially resolved information from this imagery to study patterning and heterogeneity in *Sargassum* crabs.

## Supporting Information

S1 Data Mean reflectance spectra for individual crabs and associated algae samples, with notations and crab metadata. Spectra are organized by spreadsheet with spectra in columns. The first row of all spectral spreadsheets is a header.
(XLSX)

S1 Fig Distribution of *R*(λ) values. Histograms of reflectance values at selected wavelengths corresponding to the visual sensitivities of fish and a region of close spectral matching (430 nm), a region of crab visual sensitivity [67] (490 nm), and high *R*(λ) discrepancy (660 nm) for an individual **A**) *P*. *sayi* and **B**) its associated *Sargassum* sample. Reflectance for an individual image was generally normally distributed, indicating the suitability of using mean reflectance spectra for chromatic modeling.
(TIF)

S2 Fig Illumination as downwelling irradiance *E*_*d*_(λ) at the surface (solid line) and at 15cm depth (dashed line). Irradiances are very similar in blue and green (400–550 nm) wavelengths, but diverge at longer wavelengths due to preferential attenuation and transmission across the air-sea interface.
(TIF)

S3 Fig Diffuse attenuation (*K*_*d*_) used in predator visual models for Bermuda (solid line) and Florida (dashed line) water types. Attenuation for Bermuda waters is low and characteristic of clear oceanic waters, with attenuation increasing exponentially in the red wavelengths. Florida waters attenuate much more strongly, particularly in the blue, due to the presence of colored dissolved organic matter (CDOM) and sediments.
(TIF)

S4 Fig Impact of water column on target signal (*R*(λ)*I(λ)) for a sample *P*.*sayi* and associated *Sargassum* in Bermuda water type. Attenuation with distance decreased the difference between reflected light from crab and algae that is available to the observer, modeled here for the avian predator at 1 and 5 m depth. This is particularly true in the highly absorbed red wavelengths, where the spectral signatures of both animal and background converge.
(TIF)

## References

[1] Cott HB (1940) Adaptive coloration in animals. Oxford University Press, New York

[2] Gilerson AA, Stepinski J, Ibrahim AI, You Y, Sullivan JM, Twardowski MS, et al (2013) Benthic effects on the polarization of light in shallow waters. Applied Optics 52: 8685–8700 10.1364/AO.52.008685 24513934

[3] Brady P, Travis K, Maginnis T, Cummings ME (2013) The polaro-cryptic mirror: a biological adaptation for open-ocean camouflage. Proc Natl Acad Sci USA 110: 9764–9769 10.1073/pnas.1222125110 23716701 PMC3683730

[4] Stevens M, Merilaita S Eds. (2011) Animal Camouflage: Mechanisms and Function. Cambridge University Press, Cambridge

[5] Crozier WJ (1918) Note on the Coloration of *Planes minutus* . Am Nat 52, 262–263

[6] Endler JA (1990) On the measurement and classification of colour in studies of animal colour patterns. Biol J Linn Soc 41: 315–352

[7] Hanlon RT, Chiao CC, Mathger LM, Barbosa A, Buresch KC, Chubb C (2009) Cephalopod dynamic camouflage: bridging the continuum between background matching and disruptive coloration. Phil Trans Roy Soc B 364: 429–437 19008200 10.1098/rstb.2008.0270 PMC2674088

[8] Hemmi JM, Marshall J, Pix W, Vorobyev M, Zeil J (2006) The variable colours of the fiddler crab *Uca vomeris* and their relation to background and predation. J Exp Biol 209: 4140–4153 17023607 10.1242/jeb.02483

[9] Hitchcock HB (1941) The coloration and color changes of the Gulf-Weed Crab, *Planes minutus* . Biol Bull 80: 26–30

[10] Hultgren KM, Stachowicz JJ (2008) Alternative camouflage strategies mediate predation risk among closely related co-occurring kelp crabs. Oecologia 155: 519–528 18084779 10.1007/s00442-007-0926-5

[11] Cheney KL, Skogh C, Hart NS, Marshall NJ (2009) Mimicry, colour forms and spectral sensitivity of the bluestriped fangblenny, *Plagiotremus rhinorhynchos* . Proc R Soc B 276: 1565–1573 10.1098/rspb.2008.1819 19324827 PMC2660993

[12] Barnard ME, Strandburg-Peshkin A, Yarett IR, Merz RA (2012) The blue streak: a dynamic trait in the mud fiddler crab, *Uca pugnax* . Invertebr Biol 131: 52–60

[13] Detto T, Hemmi JM, Backwell PRY (2008) Colouration and colour changes of the fiddler crab, *Uca capricornis*: a descriptive study. PLoS ONE 3(2): e1629 10.1371/journal.pone.0001629 18286186 PMC2229841

[14] Josef N, Amodio P, Fiorito G, Shashar N (2012) Camouflaging in a complex environment—octopuses use specific features of their surroundings for background matching. PLoS ONE 7: e37579 10.1371/journal.pone.0037579 22649542 PMC3359305

[15] Silbiger N and Munguia P (2008) Carapace color change in *Uca pugilator* as a response to temperature. J Exp Mar Biol Ecol 355: 41–46

[16] Stevens M, Rong CP, Todd PA (2013) Colour change and camouflage in the horned ghost crab *Ocypode ceratophthalmus* . Biol J Linn Soc 109: 257–270

[17] Stevens M, Lown AE, Wood LE (2014) Colour change and camouflage in juvenile shore crabs *Carcinus maenas* . Front Ecol Evol 2: 14

[18] Troscianko J, Stevens M (2015). Image Calibration and Analysis Toolbox—a free software suite for objectively measuring reflectance, colour, and pattern. Methods Ecol Evol 10.1111/2041-210X.12439 PMC4791150 27076902

[19] Akkaynak D, Allen JJ, Mäthger LM, Chiao CC, Hanlon RT (2013) Quantification of cuttlefish (*Sepia officinalis*) camouflage: a study of color and luminance using in situ spectrometry. J Comp Physiol A 199: 211–225 10.1007/s00359-012-0785-3 23254307

[20] Baldwin J, Johnsen S (2012) The male blue crab, *Callinectes sapidus*, uses both chromatic and achromatic cues during mate choice. J Exp Biol 215: 1184–1191 10.1242/jeb.067512 22399664

[21] Cummings ME, Jardao JM, Cronin TW, Oliveira RF (2008) Visual ecology of the fiddler crab, *Uca tangeri*: effects of sex, viewer and background on conspicuousness. Anim Behav 75:175–188

[22] Hanlon RT, Chiao CC, Mathger LM, Marshall NJ (2013) A fish-eye view of cuttlefish camouflage using in situ spectrometry. Biol J Linnean Soc 109: 535–551

[23] Chiao CC, Wickiser JK, Allen JJ, Genter B, Hanlon RT (2011) Hyperspectral imaging of cuttlefish camouflage indicates good color match in the eyes of fish predators. Proc Natl Acad Sci U S A. 108: 9148–9153 10.1073/pnas.1019090108 21576487 PMC3107294

[24] Manolakis D, Marden D, Shaw GA (2003) Hyperspectral image processing for automatic target detection applications. IEEE Signal Processing Magazine, 14: 79–116

[25] Pinto F, Mielewczik M, Liebisch F, Walter A, Greven H, Rascher U (2013) Non-invasive measurement of frog skin reflectivity in high spatial resolution using a dual hyperspectral approach. PLoS ONE 8(9): e73234 10.1371/journal.pone.0073234 24058464 PMC3776832

[26] Endler JA, Mielke PW (2005) Comparing entire colour patterns as birds see them. Biol J Linnean Soc, 86: 405–431

[27] Butler JN, Morris BF, Cadwallader J, Stoner AW (1983) Studies of *Sargassum* and the *Sargassum* community. Bermuda Biological Station Research, *Special Publication* 22: 1–307

[28] Gower JFR, Hu CM, Borstad G, King S (2006) Ocean color satellites show extensive lines of floating *Sargassum* in the Gulf of Mexico. IEEE T. Geosci Remote Sens 44: 3619–3625

[29] Parr AE (1939) Quantitative observations on the pelagic *Sargassum* vegetation of the western North Atlantic. Bull Bingham Oceanogr Collect 6: 1–94

[30] Peres JM (1982) Specific pelagic assemblages In: Kinne O, editor. Marine ecology, vol. 5, pt. 1, John Wiley and Sons, New York pp. 314–372

[31] Rooker JR, Turner JP, Holt SA (2006) Trophic ecology of *Sargassum*-associated fishes in the Gulf of Mexico determined from stable isotopes and fatty acids. Mar Ecol Prog Ser 313: 249–259

[32] Coston-Clements L, Settle LR, Hoss DE, Cross FA (1991) Utilization of the *Sargassum* habitat by marine invertebrates and vertebrates—A review. NOAA Tech Mem NMFS-SEFSC-296

[33] Dooley JK (1972) Fishes associated with the pelagic *Sargassum* complex, with a discussion of the *Sargassum* community. Contrib Mar Sci 16: 1–32

[34] Fine ML (1970) Faunal variation on pelagic *Sargassum* . Mar Biol 7: 112–122

[35] Morris BF, Mogelberg DD (1973) Identification manual to the pelagic *Sargassum* fauna Bermuda Biological Station for Research, St. George’s West

[36] Weis JS (1968) Fauna associated with pelagic *Sargassum* in the Gulf Stream. Am Midl Nat 80: 554–558

[37] Brown FA (1939) The coloration and color changes of the Gulf-Weed Shrimp, *Latreutes fucorum* . Amer Nat 73: 564–568

[38] Hacker SD, Madin LP (1991) Why habitat architecture and color are important to shrimps living in pelagic *Sargassum*: use of camouflage and plant-part mimicry. Mar Ecol Prog Ser 70: 143–155

[39] Jobe CF, Brooks WR (2009) Habitat selection and host location by symbiotic shrimps associated with *Sargassum* communities: The role of chemical and visual cues. Symbiosis 49: 77–85

[40] Moser ML, Lee DS (2012) Foraging over *Sargassum* by Western North Atlantic Seabirds. Wilson J Ornithol 124: 66–72

[41] Wells RJD, Rooker JR (2009) Feeding ecology of pelagic fish larvae and juveniles in slope waters of the Gulf of Mexico. J Fish Biol 75:1719–1732 10.1111/j.1095-8649.2009.02424.x 20738644

[42] Brooks WR, Hutchinson KA, Tolbert MG (2007) Pelagic *Sargassum* mediates predation among symbiotic fishes and shrimps. Gulf of Mexico Sci 2: 144–152

[43] Palko BJ, Beardsley GL, Richards W (1982) Synopsis of the Biolological Data on Dolphin-Fishes, *Coryphaena hippurus* Linnaeus and *Coryphaena equiselis* Linnaeus. FAO Fisheries Synopsis (130); NOAA Technical Report NMFS Circular (443)

[44] Dierssen HM, Chlus A, Russell B (2015) Hyperspectral discrimination of floating mats of seagrass wrack and the macroalgae *Sargassum* in coastal waters of Greater Florida Bay using airborne remote sensing. Remote Sens Environ 10.1016/j.rse.2015.01.027

[45] Chronin TW, Johnsen S, Marshall JN, Warrant EJ (2014) Visual Ecology. Princeton University Press, Princeton 10.1186/1472-6785-14-14

[46] Vorobyev M, Osorio D (1998) Receptor noise as a determinant of colour thresholds. Proc R Soc B 265: 351–358 9523436 10.1098/rspb.1998.0302 PMC1688899

[47] Munz FW, McFarland WN (1975) Part I. Presumptive cone pigments extracted from tropical marine fishes. Vision Res 15:1045–1062 1166604 10.1016/0042-6989(75)90001-2

[48] Fritsches KA, Partridge JC, Pettigrew JD, Marshall NJ (2000). Colour vision in billfish. Phil Trans R Soc B 355, 1253–1256 11079409 10.1098/rstb.2000.0678 PMC1692849

[49] Hart NS (2001) Variations in cone photoreceptor abundance and the visual ecology of birds. J Comp Physiol A 187: 685–697 11778831 10.1007/s00359-001-0240-3

[50] Hart NS (2004) Microspectrophotometry of visual pigments and oil droplets in a marine bird, the wedge-tailed shearwater *Puffinus pacificus*: topographic variations in photoreceptor spectral characteristics. J Exp Biol 207: 1229–1240 14978063 10.1242/jeb.00857

[51] Hart NS, Hunt DM (2007) Avian visual pigments: Characteristics, spectral tuning, and evolution. Am Nat 169: S7–S26. 10.1086/510141 19426092

[52] Langmore N, Stevens M, Maurer G (2011) Visual mimicry of host nestlings by cuckoos, Proc R Soc B 278: 2455–2463 10.1098/rspb.2010.2391 21227972 PMC3125623

[53] Hurtado-Gonzales JL, Loew E, Uy JAC. (2014) Variation in the visual habitat may mediate the maintenance of color polymorphism in a poeciliid fish. PLoS One 9 (7): e101497 10.1371/journal.pone.0101497 24987856 PMC4079317

[54] Siddiqi A, Cronin TW, Loew ER, Vorobyev M, Summers K (2004) Interspecific and intraspecific views of color signals in the strawberry poison frog *Dendrobates pumilio* . J Exp Biol 207: 2471–2485 15184519 10.1242/jeb.01047

[55] Stevens M (2007) Predator perception and the interrelation between different forms of protective coloration. Proc R Soc B 274: 1457–1464 17426012 10.1098/rspb.2007.0220 PMC1950298

[56] McPherson ML, Hilll VJ, Zimmerman RC, Dierssen HM (2011) The optical properties of Greater Florida Bay: Implications for seagrass abundance. Estuar Coasts 34: 1150–1160

[57] Mobley CD (1994) Light and Water: Radiative Transfer in Natural Waters. Academic Press, San Diego.

[58] Barbosa P, Castellanos I Eds. (2005) Ecology of predator-prey interactions. Oxford University Press, New York.

[59] Russell BJ and Dierssen HM (2012) Hyperspectral Imaging as a Tool for Camouflage Evaluation of the Sargassum Crab *Portunus sayi*. Ocean Optics Conference XXI. October 8–12. Glasgow, Scotland

[60] Darnell MZ (2012) Ecological physiology of the circadian pigmentation rhythm in the fiddler crab *Uca panacea* . J Exp Mar Biol Ecol 426–427: 39–47

[61] Green JP (1963) An analysis of morphological color change in two species of brachyuran crustaceans. Ph.D. Thesis, University of Minnesota, Minneapolis

[62] Hultgren KM, Stachowicz JJ (2012) Camouflage in decorator crabs: integrating ecological, behavioural and evolutionary approaches In: Stevens M, Merlaita S, editors. Animal Camouflage: Mechanisms and Function. Cambridge University Press, Cambridge

[63] Iampietro PJ (1999) Distribution, diet, and pigmentation of the northern kelp crab, *Pugettia producta* (Randall) in central California kelp forests. Master’s thesis, California State University, Stanislaus

[64] Smith KL (1973) Energy transformations by the Sargassum fish, *Histrio histrio* (Linnaeus). J Exp Mar Biol Ecol 12: 219–227

[65] Hutchinson KA (2004) Prey selectivity of the fishes *Stephanolepis hispidus* and *Histrio histrio* on the *Sargassum* shrimps *Latreutes fucorum* and *Leander tenuicornis*. MS Thesis, Florida Atlantic University

[66] Bursey CR (1984) Color recognition by the blue crab, *Callinectes Sapidus* Rathbun (Decapoda, Brachyura). Crustaceana 47: 278–284

[67] Detto T (2007) The fiddler crab *Uca mjoebergi* uses colour vision in mate choice. Proc Roy Soc B 274: 2785–2790 10.1098/rspb.2007.1059 PMC3227134 17848366

[68] Hyatt GW (1975) Physiological and behavioral evidence for color discrimination by fiddler crabs (Brachyura, Ocypodidae, genus *Uca*) In: Vernberg FJ, editor. Physiological ecology of estuarine organisms. University of South Carolina Press, Columbia

[69] West L (2012) Habitat location and selection by the Sargassum Crab *Portunus sayi*: The role of sensory cues. Master’s Thesis, Florida Atlantic University

[70] Johnsen S, Kelber A, Warrant E, Sweeney AM, Widder EA, Lee RL Jr, et al (2006) Crepuscular and nocturnal illumination and its effects on color perception by the nocturnal hawkmoth *Deilephila elpenor* . J Exp Biol 209: 789–800 16481568 10.1242/jeb.02053

[71] Chang GC, Dickey TD (2004) Coastal ocean optical influences on solar transmission and radiant heating rate. J Geophys Res 109, 10.

[72] Théry M, Casas J (2002) Predator and prey views of spider camouflage. Nature 415: 133 10.1038/415133a 11805822

[73] Defrize J, Théry M, Casas J (2010) Background colour matching by a crab spider in the field: a community sensory ecology perspective. J Exp Biol 213: 1425–1435 10.1242/jeb.039743 20400626

[74] Monahan EC, Mac Niocaill G (1986) Oceanic Whitecaps: And Their Role in Air-Sea Exchange Processes. Springer Science & Business Media

[75] Randolph K, Dierssen HM, Twardowski M, Cifuentes-Lorenzen A, Zappa CJ (2014) Optical measurements of small deeply penetrating bubble populations generated by breaking waves in the Southern Ocean. J Geophys. Res Oceans 119: 757–756

