# Supplementary figures and images for: Use of Hyperspectral Imagery to Assess Cryptic Color Matching in Sargassum Associated Crabs
Source: PLoS One. 2015 Sep 9;10(9):e0136260. doi: 10.1371/journal.pone.0136260 (PMC4564216; doi:10.1371/journal.pone.0136260)

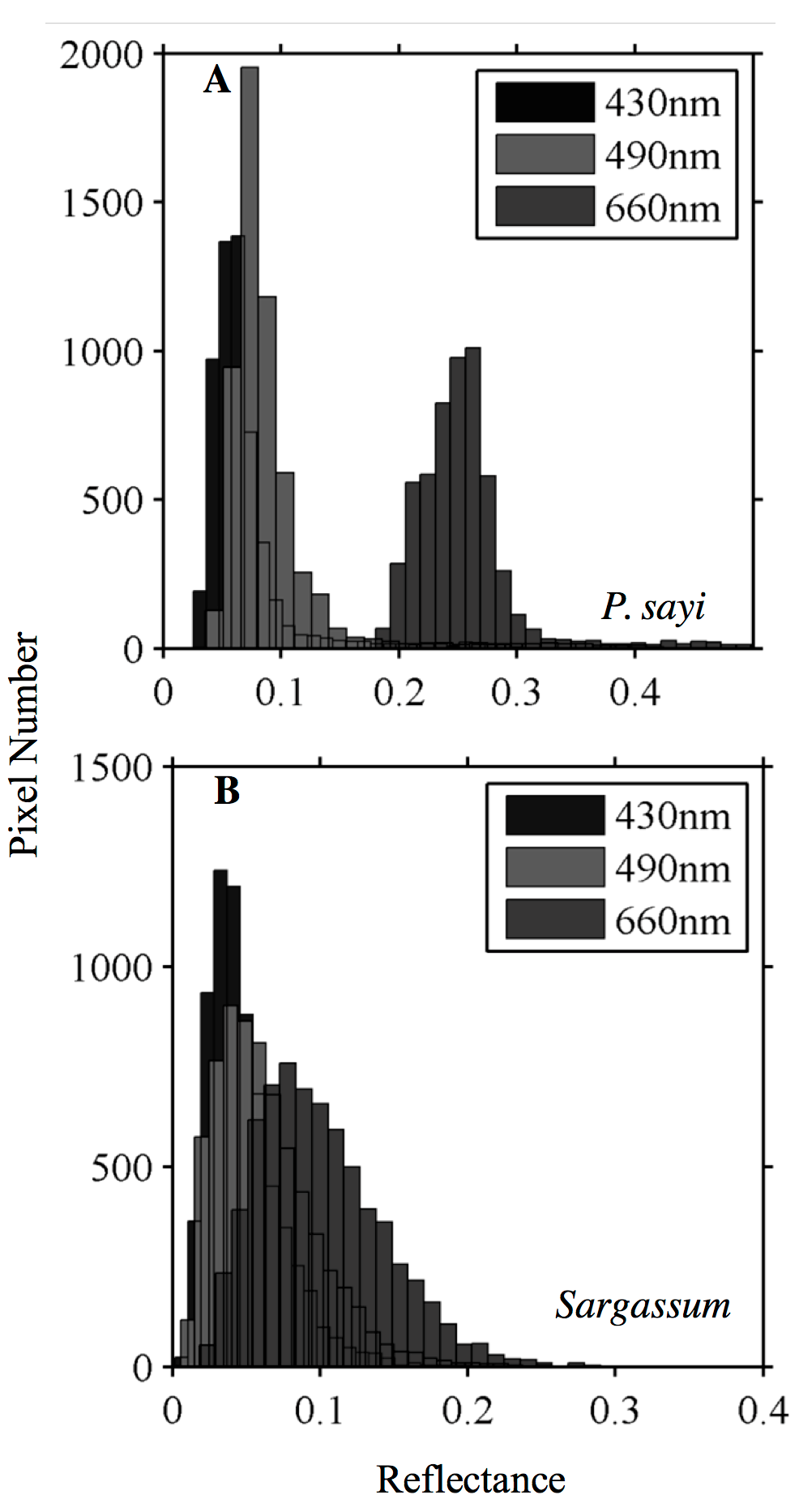

Supplement: S1 Fig — Histograms of reflectance values at selected wavelengths corresponding to the visual sensitivities of fish and a region of close spectral matching (430 nm), a region of crab visual sensitivity [67] (490 nm), and high R(λ) discrepancy (660 nm) for an individual A) P. sayi and B) its associated Sargassum sample. Reflectance for an individual image was generally normally distributed, indicating the suitability of using mean reflectance spectra for chromatic modeling. (TIF) [file pone.0136260.s002.tif]

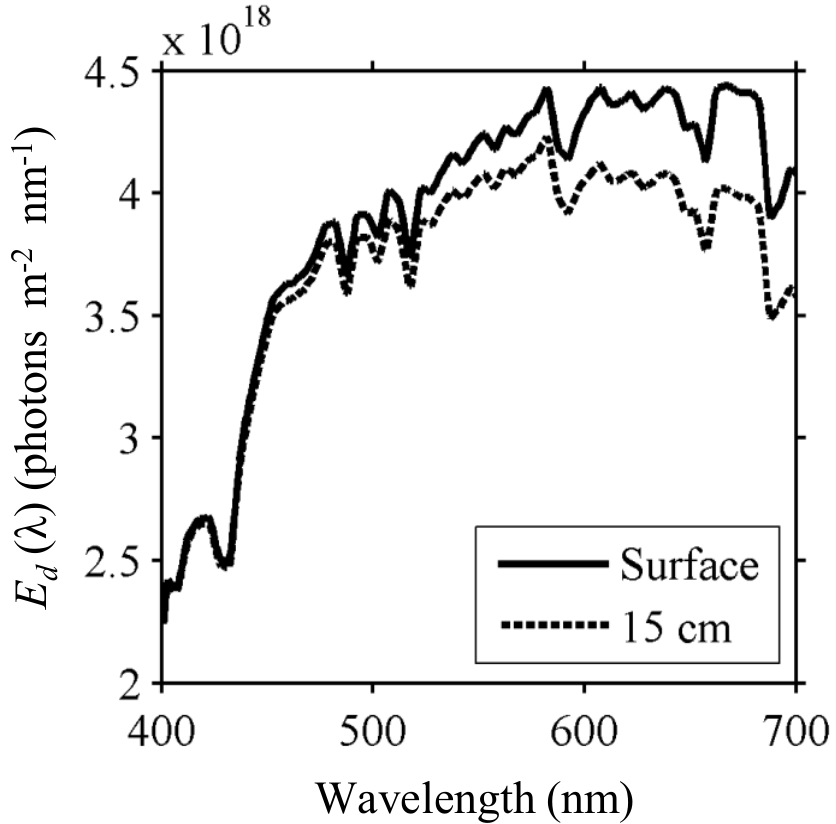

Supplement: S2 Fig — Irradiances are very similar in blue and green (400–550 nm) wavelengths, but diverge at longer wavelengths due to preferential attenuation and transmission across the air-sea interface. (TIF) [file pone.0136260.s003.tif]

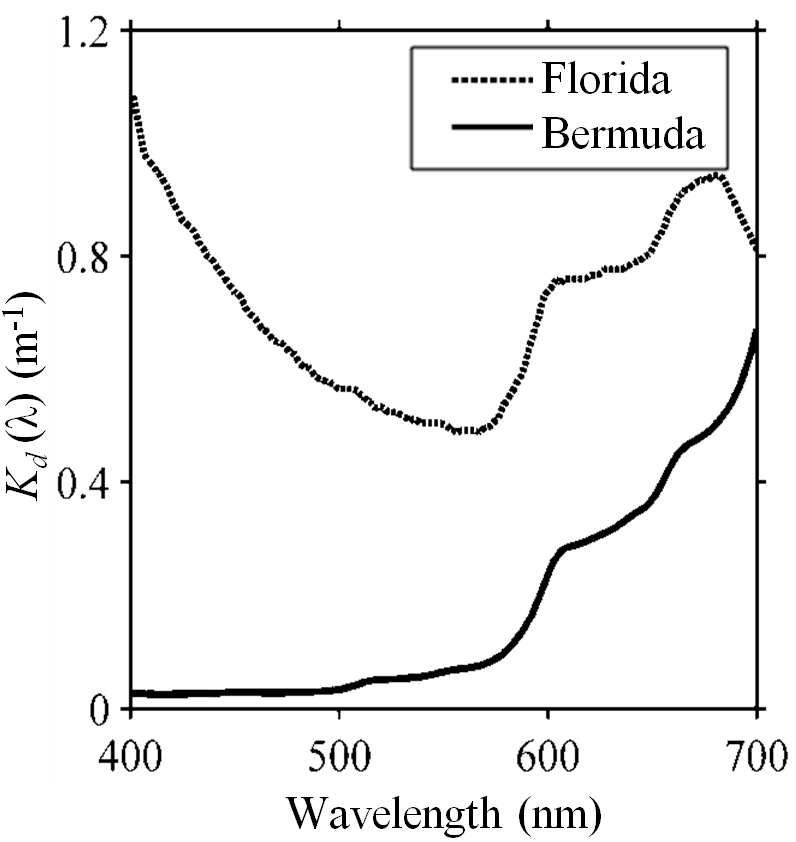

Supplement: S3 Fig — Attenuation for Bermuda waters is low and characteristic of clear oceanic waters, with attenuation increasing exponentially in the red wavelengths. Florida waters attenuate much more strongly, particularly in the blue, due to the presence of colored dissolved organic matter (CDOM) and sediments. (TIF) [file pone.0136260.s004.tif]

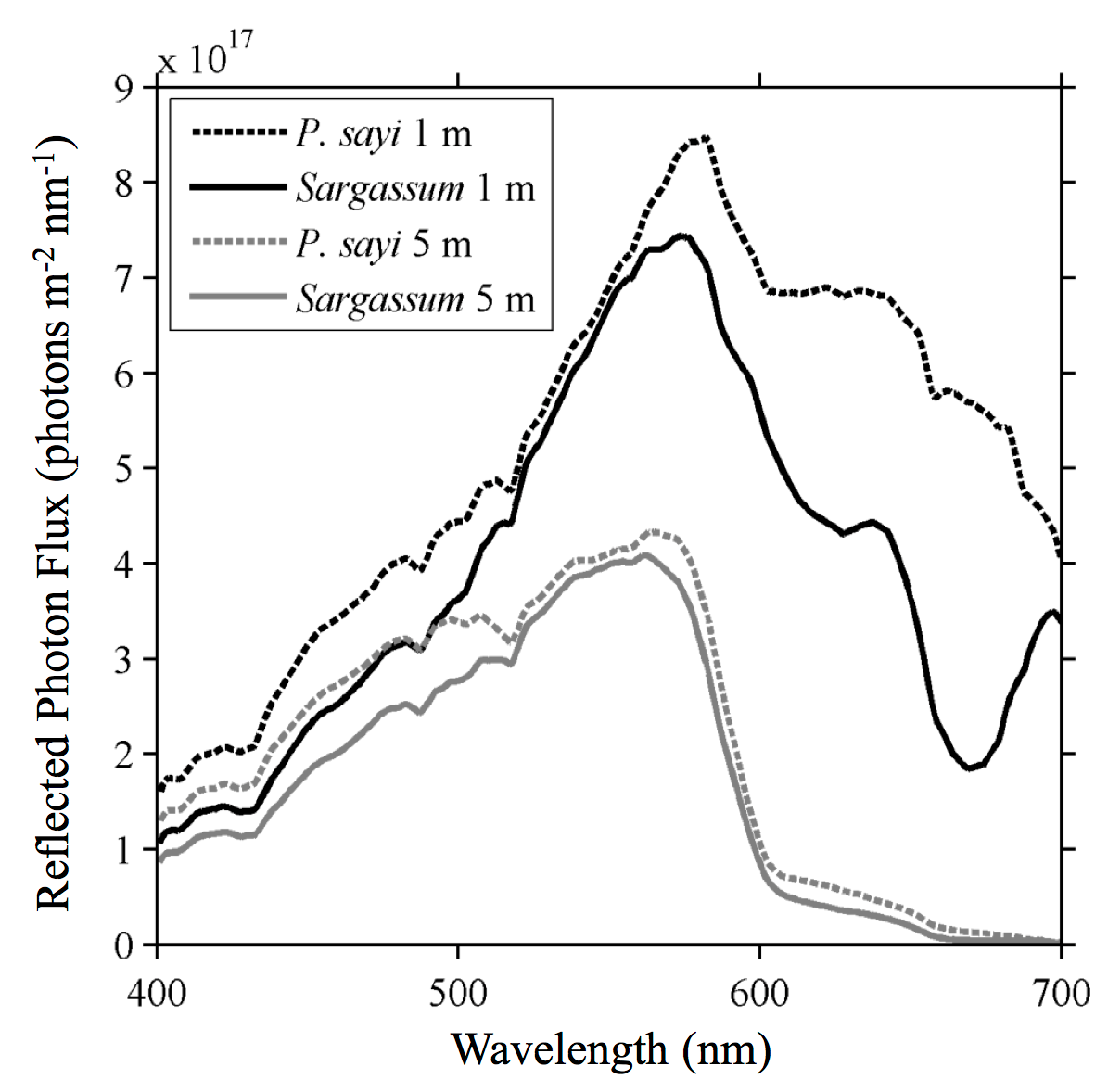

Supplement: S4 Fig — Attenuation with distance decreased the difference between reflected light from crab and algae that is available to the observer, modeled here for the avian predator at 1 and 5 m depth. This is particularly true in the highly absorbed red wavelengths, where the spectral signatures of both animal and background converge. (TIF) [file pone.0136260.s005.tif]
